# Liver‐Inspired Polyetherketoneketone Scaffolds Simulate Regenerative Signals and Mobilize Anti‐Inflammatory Reserves to Reprogram Macrophage Metabolism for Boosted Osteoporotic Osseointegration

**DOI:** 10.1002/advs.202302136

**Published:** 2023-07-03

**Authors:** Hao Gu, Yuhui Zhu, Jiawei Yang, Ruixue Jiang, Yuwei Deng, Anshuo Li, Yingjing Fang, Qianju Wu, Honghuan Tu, Haishuang Chang, Jin Wen, Xinquan Jiang

**Affiliations:** ^1^ Department of Prosthodontics Shanghai Ninth People's Hospital Shanghai Jiao Tong University School of Medicine College of Stomatology Shanghai Jiao Tong University National Center for Stomatology National Clinical Research Center for Oral Diseases Shanghai Key Laboratory of Stomatology Shanghai Research Institute of Stomatology Shanghai Engineering Research Center of Advanced Dental Technology and Materials Shanghai 200125 China; ^2^ Stomatological Hospital of Xiamen Medical College Xiamen Key Laboratory of Stomatological Disease Diagnosis and Treatment Xiamen Fujian 361008 China; ^3^ State Key Laboratory of Advanced Optical Communication Systems and Networks School of Physics and Astronomy Shanghai Jiao Tong University Shanghai 200240 China; ^4^ Shanghai Institute of Precision Medicine Shanghai Ninth People's Hospital Shanghai Jiao Tong University School of Medicine Shanghai 200125 China

**Keywords:** arginase‐2, biomimetic surface modification, macrophage metabolic reprogramming, osseointegration, polyetherketoneketone

## Abstract

Tissue regeneration is regulated by morphological clues of implants in bone defect repair. Engineered morphology can boost regenerative biocascades that conquer challenges such as material bioinertness and pathological microenvironments. Herein, a correlation between the liver extracellular skeleton morphology and the regenerative signaling, namely hepatocyte growth factor receptor (MET), is found to explain the mystery of rapid liver regeneration. Inspired by this unique structure, a biomimetic morphology is prepared on polyetherketoneketone (PEKK) via femtosecond laser etching and sulfonation. The morphology reproduces MET signaling in macrophages, causing positive immunoregulation and optimized osteogenesis. Moreover, the morphological clue activates an anti‐inflammatory reserve (arginase‐2) to translocate retrogradely from mitochondria to the cytoplasm due to the difference in spatial binding of heat shock protein 70. This translocation enhances oxidative respiration and complex II activity, reprogramming the metabolism of energy and arginine. The importance of MET signaling and arginase‐2 in the anti‐inflammatory repair of biomimetic scaffolds is also verified via chemical inhibition and gene knockout. Altogether, this study not only provides a novel biomimetic scaffold for osteoporotic bone defect repair that can simulate regenerative signals, but also reveals the significance and feasibility of strategies to mobilize anti‐inflammatory reserves in bone regeneration.

## Graphical abstract

Bioinspired by the liver extracellular skeleton morphology, a functional polyetherketoneketone scaffold is prepared that reproduces similar regenerative signaling, causing positive immunoregulation and osteogenesis. The morphology activates an anti‐inflammatory reserve (arginase‐2) to translocate retrogradely from mitochondria to the cytoplasm, reprogramming metabolism of energy and arginine. This study provides a novel biomimetic scaffold and an effective strategy for osteoporotic bone defect repair.

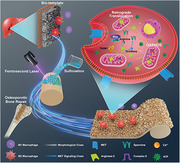

## 1 Introduction

Currently, designing efficient strategies to repair bone defects caused by trauma and diseases still faces enormous challenges.^[^ ^1^ ^]^ In the field of tissue engineering, the application of bone substitute materials, such as biological scaffolds, represents a promising approach to repairing defects.^[^ ^2^ ^]^ Compared with metal or ceramic materials, polyetherketoneketone (PEKK) is considered a desirable candidate for bone tissue engineering due to its excellent mechanical properties, which are more consistent with the prerequisites for biological scaffolds.^[^ ^3^ ^]^ However, its inherent bioinertness impairs physiological regenerative biocascades, leading to intense inflammatory responses and osteoblast dysfunction.^[^ ^4^ ^]^ Therefore, a deliberate strategy needs to be designed to overcome the limitations.

To date, strategies to modify PEKK, a member of polyaryletherketones (PAEKs), have mainly focused on adding bioactive particles to a prepared porous surface to steer cell fates.^[^ ^5^ ^]^ Nevertheless, concerns, including burst release and effective dose deviation, require attention.^[^ ^6^ ^]^ Thus, the concept of tissue‐inducing biomaterials has been introduced, defined as a biomaterial designed to induce the regeneration of damaged tissue without adding exogenous cells or bioactive factors.^[^ ^7^ ^]^ However, conventional modification designs on PEKK and other similar PAEKs by constructing porous structures using sulfonation have not been successful to orchestrate regenerative biocascades.^[^ ^3^, ^8^ ^]^ The morphological clues regulating tissue regeneration need to be further explored.

Incorporating the wisdom of the natural morphological clues of organisms, biomimetic strategies have shown unique superiority in the design of scaffold materials for tissue repair.^[^ ^5^, ^9^ ^]^ However, solely imitating the physical morphology and disregarding the transformation of intrinsic biological signals is insufficient. It is of greater significance to reproduce the corresponding biological functions to achieve functional bionics via the recurrence of morphological clues. Among natural tissues, liver tissue exhibits outstanding regenerative ability. Many growth factors, most notably hepatocyte growth factor (HGF), play important roles in tissue regeneration.^[^ ^10^ ^]^ MET, known as the HGF receptor, is required for various morphogenetic bioevents and promotes tissue remodeling, such as bone regeneration, which underlies wound repair and organ homeostasis.^[^ ^11^ ^]^ MET signaling also plays a crucial role in the organization and transformation of external signals into intracellular biochemical information.^[^ ^11^ ^]^ In addition to its multistage blood supply, the extracellular skeleton structure of the liver may explain the rapid immune response and tissue repair.^[^ ^12^ ^]^ These findings suggest a potential synergistic relationship between the liver skeleton structure and MET signaling, which is worth studying and applying to improve bone tissue repair.

Macrophages, as pioneers of the immune response and regeneration, can modulate the microenvironment in response to morphological clues on the implanted material surface to pave the way for subsequent tissue repair.^[^ ^13^ ^]^ Material bioinertness and improper morphological clues will disrupt regenerative immune orchestration, leading to delayed bone healing.^[^ ^14^ ^]^ The negative effect is particularly evident in complicated pathological microenvironments, such as osteoporosis.^[^ ^15^ ^]^ Yet osteoporosis poses higher requirements for optimal mechanical properties, deepening the contradiction between the need for and concerns about PEKK application.^[^ ^16^ ^]^ In addition, a prolonged inflammatory and pathological milieu can weaken the original anti‐inflammatory systems, such as arginase‐1 (Arg1), attenuating the effect of exogenous immunoregulation.^[^ ^17^ ^]^ Arginase‐2 (Arg2), different from Arg1, can serve as a reserve and reprogram arginine metabolism in an inflammatory microenvironment to resurge macrophage state.^[^ ^18^ ^]^ Moreover, macrophages are modulated by MET signaling, which is influenced by designated morphological clues and can be pertinently reprogrammed to orchestrate immunoregulation and tissue regeneration.^[^ ^11^ ^]^ Given this, an engineered design derived from liver morphological clues and a rational utilization of Arg2 can endow PEKK with biological regulation ability and revitalize its clinical application.

**Scheme 1:**
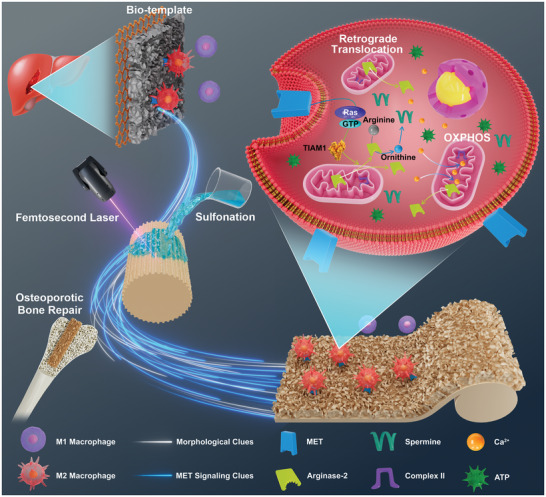
Schematic diagram showing the biomimetic functionalization design for liver‐inspired PEKK scaffolds and the mechanism of macrophage metabolic reprogramming in osteoporotic bone regeneration via mobilization of anti‐inflammatory reserves.

Herein, we found that the liver extracellular skeleton structure could effectively activate MET signaling to regulate macrophage polarization. Inspired and guided by this unique morphological bio‐template, a biomimetic surface structure was designed and prepared on PEKK scaffolds via femtosecond laser etching and sulfonation. The biomimetic design reproduced MET signaling in macrophages and conferred bioinert PEKK with biological functionalization, orchestrating regenerative immunoregulation and optimizing osteogenesis. MET signaling of biomimetic morphology mediates retrograde translocation of Arg2 from mitochondria into the cytoplasm, reprogramming energy metabolism and arginine metabolism (**Scheme** **1**). This deliberate strategy incorporates morphological signaling transduction and mobilization of the host's anti‐inflammatory reserves, showing great promise in overcoming the barriers of material bioinertness and scaffold application in an inflammatory microenvironment.

## 2 Results

### 2.1 MET Signaling Induced by the Liver Extracellular Skeleton Structure

**Figure 1:**
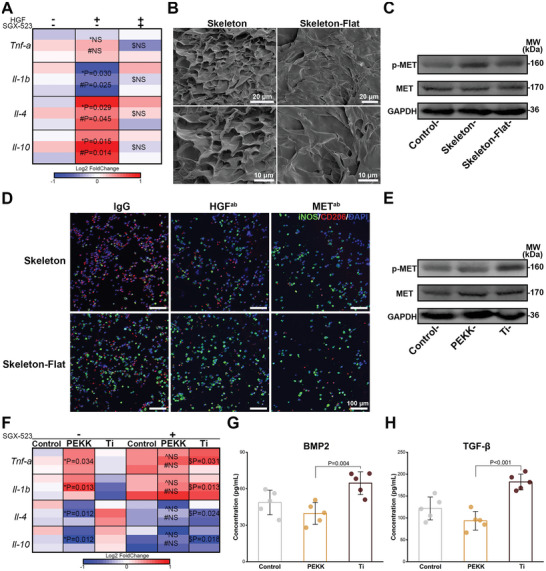
MET signaling triggered by the liver extracellular skeleton. A) Heat map depicting the expression of genes related to polarization of RAW264.7 cells stimulated by hepatocyte growth factor (HGF) and the MET inhibitor (SGX‐523) (*n* = 3; *, #, and $ represent HGF vs control, HGF vs HGF+SGX‐523, and HGF+SGX‐523 vs control). B) Scanning electron microscopy (SEM) images capturing surface of the Skeleton sample and the Skeleton‐Flat sample. C) Expression of MET and p‐MET of RAW264.7 cells cultured on the Skeleton sample and the Skeleton‐Flat sample. D) Immunofluorescent staining images of polarization markers of RAW264.7 cells cultured on two liver extracellular skeleton samples treated with HGF antibody or MET antibody for 3 days (iNOS, green; CD206, red; DAPI, blue). E) Expression of MET and p‐MET of RAW264.7 cells cultured on the PEKK scaffold and the titanium scaffold for 3 days. F) Heat map depicting the expression of genes related to polarization of RAW264.7 cells cultured on the PEKK scaffold and the titanium scaffold relative to that of the control group treated with or without SGX‐523 (n = 3; *, ^, #, and $ represent PEKK vs Ti, PEKK+SGX‐523 vs Ti+SGX‐523, PEKK vs PEKK+SGX‐523, and Ti vs Ti+SGX‐523, respectively). The concentrations of G) BMP2 and H) TGF‐*β* of the microenvironment regulated by RAW264.7 cells cultured on the PEKK scaffold and the titanium scaffold for 4 days (error bars, means ± SD; *n* = 5). Data were analyzed by ordinary one‐way ANOVA with Tukey's post‐hoc test and respective P values are provided.

MET signaling is involved in functional cooperation between different signal axes and pathways related to macrophage regenerative immunoregulation.^[^ ^11^ ^]^ When HGF activated MET signaling, RAW264.7 cells exhibited M2 polarization (**Figure** **1**A). However, this change was eliminated by the MET inhibitor (SGX‐523), indicating a strong correlation between MET signaling and M2 polarization. To explore the relationship between morphological clues and biological information in tissue regeneration, the liver extracellular skeleton structures were prepared. After decellularization (Figure S1A–C, Supporting Information) and lyophilization, the surface of the liver tissue blocks presented a 3D petal‐like unit morphology with an interval of ≈6 µm (denoted as “Skeleton”) (Figure 1B). For Skeleton‐Flat, the acellular tissue blocks were physically extruded prior to lyophilization to remove surface characteristic structures without affecting their composition (Figure S1D,E, Supporting Information). Interestingly, the characteristic structure increased p‐MET expression (Figure 1C) and promoted early immune repair transformation, showing higher CD206 (M2) and lower inducible nitric oxide synthase (iNOS, M1) expression (Figure 1D). Blockage of MET (using a MET antibody) eliminated the polarization difference between macrophages on the two skeleton samples, indicating that the unique structure mediated M2 polarization via MET signaling. It is noteworthy that macrophages on the surface of the liver skeleton still showed a preference for M2 polarization when HGF was neutralized (using HGF antibody), compared with those on the surface without the characteristic structure (Figure 1D). These findings suggested that the characteristic structure could simulate MET signaling and realize the transformation of morphological clues into biological information. Furthermore, compared to titanium, PEKK scaffolds were unable to increase the levels of MET signaling and M2 polarization of macrophages (Figure 1E,F), which might explain the intrinsic cause of PEKK bioinertness. The release of osteogenic factors by macrophages was also inhibited (Figure 1G,H). From this, we propose that one peculiar avenue to endow PEKK with biological regulation ability is to harness the morphological resources of the liver.

### 2.2 Preparation and Characterization of Biomimetic PEKK Scaffolds

**Figure 2:**
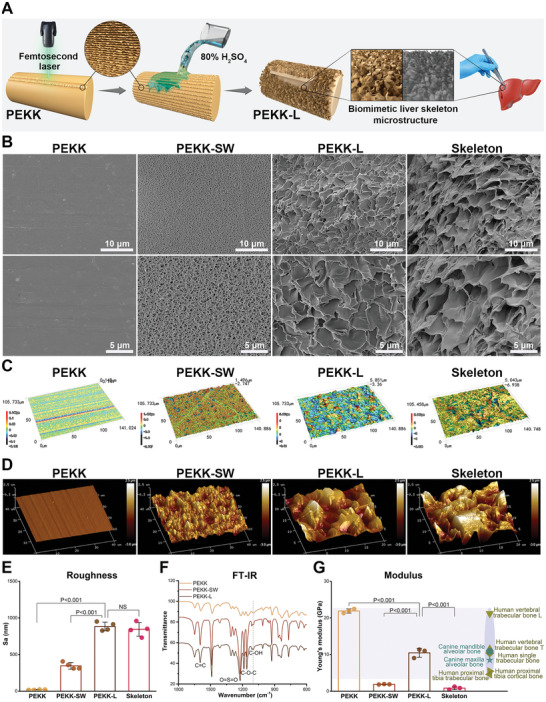
Preparation and characterization of biomimetic PEKK scaffolds. A) Schematic illustration of the preparation of PEKK‐L scaffolds. B) Scanning electron microscopy (SEM) images, C) 3D surface optical profiles and D) atomic force microscopy (AFM) images of various PEKK scaffolds and the liver extracellular skeleton. E) Surface roughness of the PEKK scaffolds and the liver extracellular skeleton detected by 3D optical profiles (error bars, means ± SD; *n* = 4). F) Fourier transform infrared (FT‐IR) spectra of various PEKK scaffolds. G) Elastic modulus of various PEKK scaffolds and the liver extracellular skeleton detected by AFM (error bars, means ± SD; *n* = 3) with elastic modulus of representative natural bone tissue according to references. Statistical significance was analyzed by ordinary one‐way ANOVA with Tukey's post‐hoc test and respective P values are provided.

Bioinspired by the liver extracellular skeleton structure and guided by this bio‐template, we constructed the characteristic structure by physical and chemical means. As shown in **Figure** **2**A, the palisade structures (8 µm apart) were prepared by femtosecond laser (Figure S2A, Supporting Information), which were further processed with 80% H_2_SO_4_ to create an independent petal‐like unit morphology on the PEKK surface (denoted as “PEKK‐L”), with hydrothermal treatment subsequently to remove residual acid. As a mainstream means of modification, sulfonating PEKK with 98% H_2_SO_4_ was included as a comparison (PEKK‐SW) in this study.^[^ ^19^ ^]^ The 3D petal‐shaped unit morphology of the PEKK‐L surface resembled the natural extracellular skeleton of the liver, which was different from the smooth surface and the microporous network of the PEKK and PEKK‐SW surfaces, respectively (Figure 2B). The lamellar prominences of the PEKK‐L surface were closely spaced ≈5 µm apart (Figure 2B), and the biomimetic modified layer was ≈30 µm thick (Figure S2B, Supporting Information). The unique morphology with microgrooves, islands, and microholes on the PEKK‐L surface was similar to that of the liver skeleton rather than the flat or single porous structure on the PEKK and PEKK‐SW surfaces (Figure 2C). PEKK‐L shared a semblable texture and undulating shape with the liver skeleton (Figure 2D). In addition, the surface roughness of the PEKK‐L (≈0.884 µm) was close to that of the liver skeleton (≈0.844 µm) (Figure 2E). The composition of PEKK was not significantly disturbed by the addition of a small amount of exogenous sulfur (only 1.08%) (Figure S2C, Supporting Information). The asymmetric tensile vibration peak of O=S=O was at 1255 cm^−1^ in the FTIR spectra (Figure 2F), indicating the formation of —SO_3_H groups.^[^ ^20^ ^]^ Moreover, the characteristic peak at 1100 cm^−1^ was attributed to C—OH groups on the PEKK‐L surface, resulting from the C—O—C bond breaking and reacting with water molecules to form C—OH groups during the laser etching process.^[^ ^21^ ^]^ The introduction of hydrophilic components (—SO_3_H groups) and active sites (—OH groups) is expected to improve the hydrophilicity of PEKK, considering the combined effects of these functional groups, as well as surface roughness and morphological clues, which can significantly influence the contact angle (Figure S2D, Supporting Information).^[^ ^5^, ^21^, ^22^ ^]^ The pH value remained largely unaffected by the introduction of the functional groups (Figure S2E, Supporting Information). Fortunately, PEKK‐L maintained the prominent mechanical properties of polymer materials, which possessed an appropriate elastic modulus (≈10.469 GPa) matching that of natural bone tissue (Figure 2G).^[^ ^23^ ^]^ The unique morphology and outstanding mechanical properties of PEKK‐L make it a promising candidate for use in biomedical engineering.

### 2.3 Biocompatibility and Simulated MET Signaling of PEKK‐L

**Figure 3:**
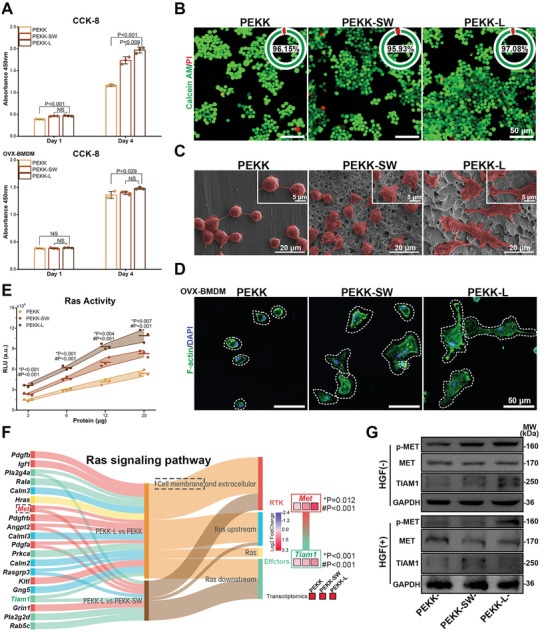
Biocompatibility and functional bionics of biomimetic PEKK scaffolds. A) CCK8 results of RAW264.7 cells and OVX‐BMDMs cultured on various scaffolds for 1 and 4 days, respectively (error bars, means ± SD; *n* = 3). B) Live/Dead fluorescent images of RAW264.7 cells on the scaffolds and the proportion of living cells in upper right corner of images (living cells, green; dead cells and red). C) SEM observations for RAW264.7 cells (pseudocoloured to red) cultured on the scaffolds for 3 days. D) Confocal laser scanning microscope observations for cytoskeleton of OVX‐BMDMs on the scaffolds for 3 days (F‐actin, green; DAPI, blue). E) Ras activity of RAW264.7 cells (*n* = 3). F) A sankey diagram obtained by RNA sequencing visualizing the differentially expressed genes (DEGs) of RAS signaling pathway with the fold change in the expression of *Met* and *Tiam1* (* and # represent PEKK‐L vs PEKK‐SW and PEKK‐L vs PEKK, respectively). G) Expression of MET, p‐MET, and TIAM1 of RAW264.7 cells cultured on various scaffolds treated with or without HGF for 3 days. Statistical significance was analyzed by ordinary one‐way ANOVA with Tukey's post‐hoc test and respective *P* values are provided.

Favorable biocompatibility is the basis of functional implanted materials. PEKK‐L exhibited excellent cell viability for both RAW264.7 cells and ovariectomized rat bone marrow‐derived macrophages (OVX‐BMDMs) (**Figure** **3**A,B). RAW264.7 cells showed a flatter morphology and a greater tendency to adhere to the PEKK‐L surface, as opposed to the globular or fusiform morphology on the surfaces of PEKK and PEKK‐SW (Figure 3C). Furthermore, F‐actin filaments of OVX‐BMDMs appeared to stretch and spread extensively on the PEKK‐L surface (Figure 3D).

Advanced biomimetic strategies are not confined to the mere imitation of physical morphology, but can utilize the physical clues of bio‐templates to achieve functional surface.^[^ ^24^ ^]^ Therefore, we further explored the functional bionics of the prepared scaffolds at the biological level. The Ras pathway, as a classic downstream signaling pathway of MET, was efficiently activated by the biomimetic structure (Figure 3E). Additionally, the differentially expressed genes (DEGs) related to the Ras signaling pathway of macrophages on various scaffolds were displayed by RNA sequencing (Figure 3F). Clearly, different from the spatial distribution of differential genes of PEKK‐L versus PEKK, the DEGs of PEKK‐L versus PEKK‐SW predominantly consisted of cell membranes and were downstream of Ras, and the recurring genes were mainly involved in cell membrane receptors. *Met* was found to be significantly upregulated in the PEKK‐L group, as was the related downstream gene *Tiam1* (T‐lymphoma invasion and metastasis‐inducing protein 1). At the protein level, the signal transduction of the MET‐TIAM1 axis was verified in Figure 3G. Therefore, MET/Ras/TIAM1 signaling might play a role in transmitting morphological signals of the biomimetic structure into cells and activating subsequent biological processes.

### 2.4 Energy Metabolism is an Important Biological Process Regulated by the Biomimetic Structure

**Figure 4:**
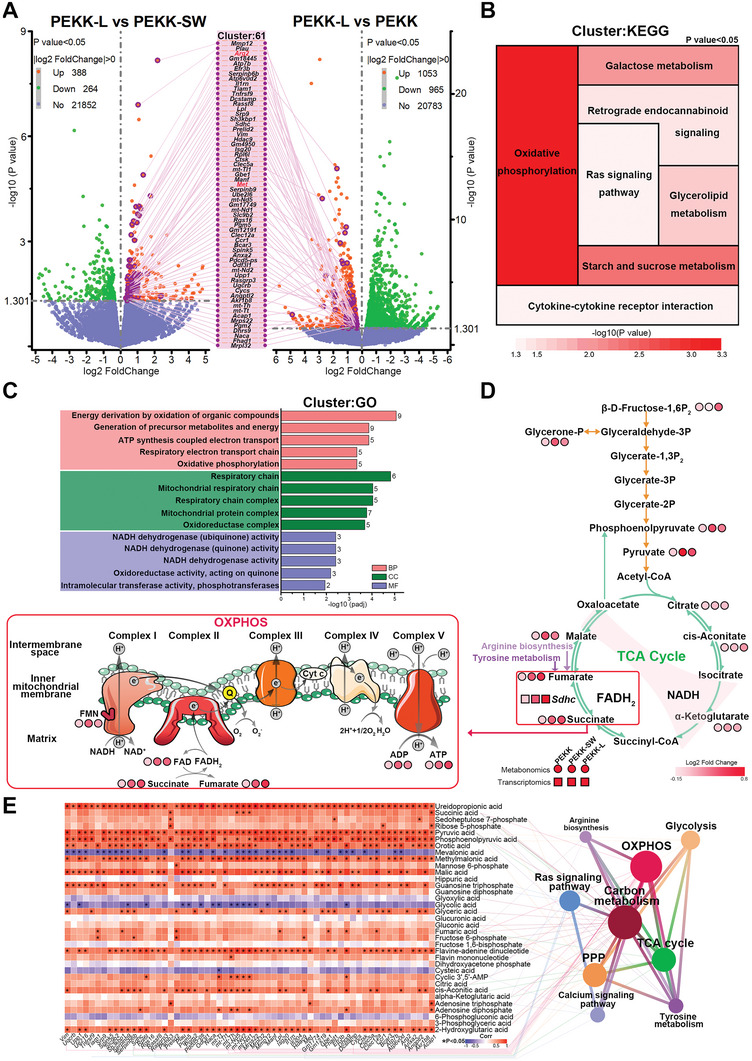
Exploration of gene expression and energy metabolism patterns and functional enrichment analysis. A) Volcano plots of DEGs of RAW264.7 cells cultured on various scaffolds for 3 days and the intersecting cluster of upregulated DEGs. B) KEGG pathway enrichment analysis and C) GO pathway enrichment analysis of the intersecting gene cluster. D) Central carbon metabolism analysis visualizing the levels of differentially expressed metabolites related to the TCA cycle and OXPHOS. E) A heat map of combined analysis of RNA sequencing and central carbon metabolism depicting the correlation between the changes in the expression of metabolites and intersecting DEGs with functional enrichment analysis of the related genes and metabolites. *n*  =  3 biological samples per group. Statistical significance was analyzed by ordinary one‐way ANOVA with Tukey's post‐hoc test and respective *P* values are provided.

To further reveal the underlying mechanism and associated bioevents during the macrophage response elicited by the biomimetic surface design, in‐depth mRNA expression profile sequencing was employed. The intersecting upregulated genes (total of 61) in PEKK‐L compared with PEKK and PEKK‐SW were analyzed, which constituted a gene cluster (**Figure** **4**A). As expected, *Met*, a key gene to the biomimetic functionalization guided by the bio‐template, appeared in the gene cluster. Seven significant pathways were enriched and analyzed by the Kyoto Encyclopedia of Genes and Genomes (KEGG) database, which mainly belonged to energy‐related carbon metabolism (Figure 4B). The Gene Ontology (GO) database enrichment analysis showed that the genes were mostly enriched in energy metabolism, including energy derivation, oxidative phosphorylation (OXPHOS), and mitochondrial complex (Figure 4C). Therefore, energy metabolism was further investigated via central carbon metabolism analysis. The detailed metabolomics analysis visually displayed marked increases in two metabolites, fumarate and succinate, which were related to succinate dehydrogenase (SDH) in the tricarboxylic acid cycle (TCA cycle). In addition, the ATP activity increased concomitantly during OXPHOS, which might be attributed to SDH, also known as complex II, the bifunctional enzyme that links the TCA cycle and electron transport chain (Figure 4D). The combined analysis of RNA sequencing and central carbon metabolism indicated that the related genes and metabolites were mainly enriched in OXPHOS, the TCA cycle, the pentose phosphate pathway, glycolysis, the calcium signaling pathway, arginine biosynthesis, and tyrosine metabolism (Figure 4E). Fumarate metabolism, regulated by SDH, is associated with arginine metabolism. Consistently, arginine metabolism and tyrosine metabolism are involved in macrophage polarization and the Ras signaling pathway, respectively.^[^ ^11^, ^25^ ^]^ Therefore, energy metabolism may act as a bridge between the Ras signaling activated by the biomimetic morphology and the ultimate polarization phenotype.

### 2.5 PEKK‐L Promoted Mitochondrial Respiration in Macrophages by Complex II

**Figure 5:**
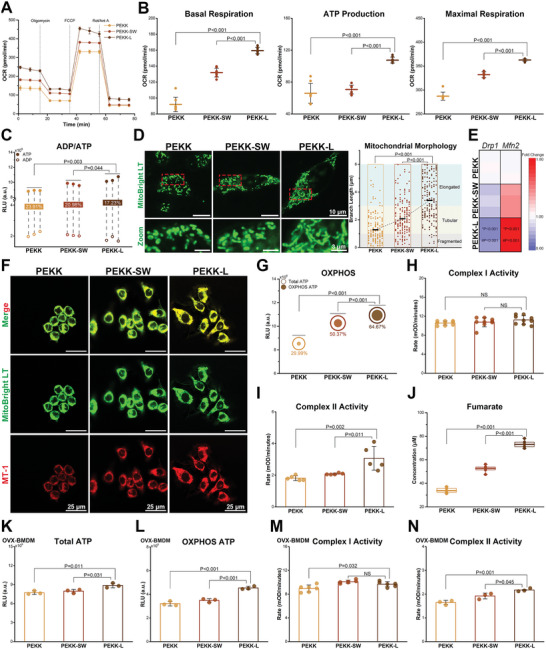
Mitochondrial respiration and mitochondrial dynamic evaluations of macrophages regulated by scaffolds. A) Oxygen consumption rates (OCR) of RAW264.7 cells cultured on scaffolds with addition of Oligomycin (1 µm), FCCP (0.9 µm) and Rotenone + Antimycin A (Rot/Ant A) (0.5 µm) sequentially (error bars, means ± SD; *n* = 8). B) Basal respiration, ATP production and maximal respiration calculated by OCR curves (error bars, means ± SD). C) The ratio of ADP to ATP (*n* = 3). D) Mitochondrial morphology of RAW264.7 cells cultured on scaffolds with mitochondrial morphology analysis. E) Heat map depicting the expression of *Drp1* and *Mfn2* (*n* = 3; * and # represent PEKK‐L vs PEKK‐SW and PEKK‐L vs PEKK, respectively). F) Mitochondrial membrane potential of RAW264.7 cells. G) Total and OXPHOS ATP (*n* = 3). Comparison of RAW264.7 cells cultured for 3 days on scaffolds for H) complex I activity (*n* = 9), I) complex II activity (*n* = 5) (error bars, means ± SD) and J) fumarate (lower and upper box boundaries, line inside box and lower and upper lines represent 25th and 75th percentiles, median, minimum, and maximum, respectively; *n* = 5). Comparison of OVX‐BMDMs cultured for 3 days on scaffolds for levels of K) total ATP (*n* = 3), L) OXPHOS ATP (*n* = 3), M) complex I activity (*n* = 6) and N) complex II activity (*n* = 3; error bars, means ± SD). Data were analyzed by ordinary one‐way ANOVA with Tukey's post‐hoc test and respective *P* values are provided.

Given the considerable differences in bioinformation analysis in energy metabolism, further investigation of mitochondrial respiration and dynamics was conducted. The oxygen consumption rate analysis revealed increased basal respiration, ATP production, and maximal respiration in the PEKK‐L group (**Figure** **5**A,B). In addition, a higher ATP/ADP ratio was observed (Figure 5C). Mitochondrial morphology and function vary with cell phenotype.^[^ ^26^ ^]^ Punctate and fragmented mitochondria (<1 µm) were observed in the PEKK group, resulting from excessive mitochondrial fission (Figure 5D). Conversely, abundant elongated mitochondria (>3 µm) were woven into the network in the PEKK‐L group, indicating a fusion state that is in favor of mitochondrial respiration.^[^ ^26^ ^]^ The expression of mitochondrial fusion (*Mfn2*) and fission (*Drp1*) genes was consistent with these findings (Figure 5E). The biomimetic design maximized the ability to resurge mitochondrial membrane potential and elevate OXPHOS levels (Figure 5F,G). Furthermore, the byproducts cytoplasmic reactive oxygen species (ROS) and mitochondrial ROS were effectively scavenged, resulting from the enhanced antioxidant capacity of PEKK‐L (Figure S3A–E, Supporting Information). A decrease in lactate was another example (Figure S3F, Supporting Information). Further investigation into the electron transport chain revealed that it was complex II, not complex I, that dominated the mitochondrial respiration process (Figure 5H,I), corresponding with the bioanalysis. The increase in downstream fumarate echoed the above results as well (Figure 5J). Ex vivo OVX‐BMDMs cultured on various PEKK scaffolds showed similar changes, with an increase in OXPHOS‐derived ATP and enhanced complex II activity (Figure 5K–N). These findings suggested that PEKK‐L enhanced mitochondrial respiration, specifically through complex II. The enhancement can be attributed to specific material characteristics (morphology, surface roughness, and hydrophilicity) leading to cytoskeleton rearrangement, which in turn affects network positioning and mitochondrial dynamics.^[^ ^27^ ^]^

### 2.6 PEKK‐L Induced Retrograde Translocation of Arg2 Activated by MET Signaling to Reprogram Arginine Metabolism and Energy Metabolism

**Figure 6:**
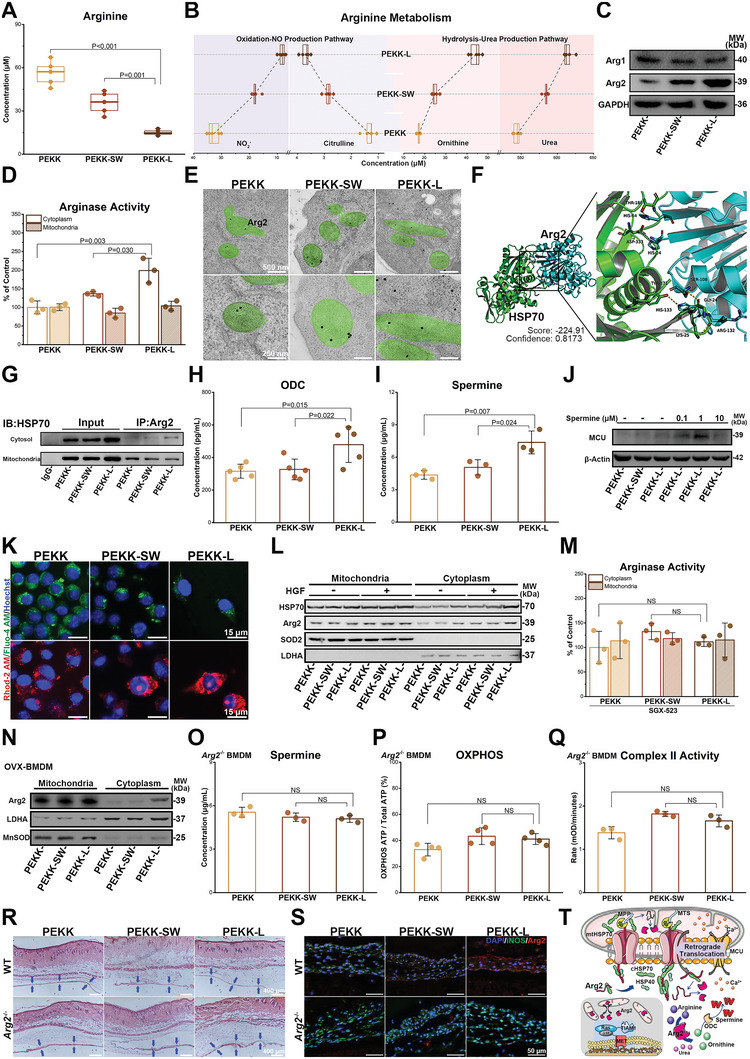
Retrograde translocation of Arg2 in macrophages on PEKK‐L to reprogram arginine metabolism and energy metabolism. RAW264.7 cells were cultured on scaffolds for levels of A) arginine concentration (*n* = 5) and B) downstream metabolite concentration (*n* = 5). C) Expression of Arg1 and Arg2. D) Arginase activity in the cytoplasm and mitochondria (*n* = 3). E) Immunoelectron microscopy of mitochondria (green) and Arg2 (labeled with immune colloidal gold). F) Proposed docking interface between Arg2 (cyan) and HSP70 (green) based on molecular docking. G) Interaction between cytosol HSP70 and immunoprecipitated Arg2. Concentration of H) ornithine decarboxylase (*n* = 5) and I) spermine (*n* = 3). J) Expression of MCU treated with or without exogenous spermine. K) Cytoplasmic Ca^2+^ (green) and mitochondrial Ca^2+^ (red) in RAW264.7 cells. L) Expression of Arg2 and HSP70 treated with or without HGF. M) Arginase activity in RAW264.7 cells cultured on scaffolds for 3 days treated with SGX‐523 (*n* = 3). N) Arg2 expression in the cytoplasm and mitochondria of OVX‐BMDMs. Comparison of O) spermine (*n* = 3), P) OXPHOS (*n* = 4), and Q) complex II activity (*n* = 3) in *Arg2* ^−/−^‐BMDMs. R) HE and S) immunofluorescent staining of fibrous layer (blue arrows) of dorsal skin in wild type and *Arg2* ^−/−^ mice after implantation for 3 days (*n* = 3). T) A schematic diagram showing the putative molecular mechanism. Data were analyzed by ordinary one‐way ANOVA with Tukey's post‐hoc test and respective *P* values are provided.

The outcome of arginine determines macrophage polarization.^[^ ^28^ ^]^ In the PEKK‐L group, more arginine was consumed by hydrolysis than oxidation, and downstream urea and ornithine were generated (**Figure** **6**A,B). However, the expression of argininosaccinate synthase‐1 (ASS1) and argininosuccinate lyase (ASL), which are the enzymes involved in arginine production, showed no significant difference among the three groups (Figure S4A,B, Supporting Information). Thus, the expression of two key enzymes in the hydrolysis of arginine (Arg1 and Arg2) was further detected. On one hand, the biomimetic design increased Arg2 expression but had no significant effect on Arg1 (Figure 6C). On the other hand, Arg2 was found in the co‐upregulated gene cluster (Figure 4A; Figure S4C, Supporting Information). Moreover, the correlation between Arg2 expression and the characteristic structure was verified at the mRNA level on the surface of the bio‐template (Figure S4D, Supporting Information). Hence, mechanisms of biological functions induced by morphological clues may lie in the orchestration between MET/Ras signaling, Arg2, and energy metabolism.

Generally, Arg2 is localized in the mitochondria with a mitochondrial targeting sequence (MTS), whereas Arg1 without MTS is in the cytoplasm.^[^ ^29^ ^]^ Nonetheless, combined with the analysis of arginase expression, a paradox arose: differences in arginase activity occurred within the cytoplasm rather than within the mitochondria (Figure 6D). It was speculated that Arg2 in macrophages on the PEKK‐L surface retrogradely translocated, similar to that in aortic endothelial cells.^[^ ^30^ ^]^ Indeed, retrograde translocation was observed in the PEKK‐L group (Figure 6E). Inspiringly, similar to fumaratase‐1, which also contains MTS, the translocation might occur through the combined action of mitochondrial and cytoplasmic chaperones.^[^ ^31^ ^]^ Molecular docking simulations showed that Arg2 might bind to heat shock protein 70 (HSP70) (Figure 6F). Co‐immunoprecipitation proved that Arg2 bound to HSP70, with more binding in the cytoplasm and less binding in mitochondria in the PEKK‐L group (Figure 6G). Mitochondrial HSP70 (mtHSP70) binds to linear protein molecules and hinges proteins into the mitochondrial matrix.^[^ ^32^ ^]^ Therefore, we hypothesize that when the linear Arg2 protein enters mitochondria, the free part in the cytoplasm is more readily bound and folded by cytoplasmic HSP70 (cHSP70), preventing the transportation of Arg2 into mitochondria induced by mtHSP70 and causing retrograde translocation (Figure 6T), though not ruling out the possibility of backcrossing from the translocation pore in mitochondrial membranes.^[^ ^31^ ^]^ Furthermore, HSP40, which is located in the cytoplasm, could also assist cHSP70 in expanding and activating functional domains (Figure S4E, Supporting Information).^[^ ^33^ ^]^ Increased HSP40 protein expression was observed in the PEKK‐L group, favoring retrograde translocation (Figure S4F, Supporting Information). The phenomenon of retrograde translocation was also found in OVX‐BMDMs (Figure 6N).

Subsequently, Arg2 generated more ornithine in the cytoplasm (Figure 6B), along with higher expression of ornithine decarboxylase (ODC), causing an increase in spermine (Figure 6H,I). Spermine has a strong affinity for anions, resulting in more free Ca^2+^ in the cytoplasm.^[^ ^34^ ^]^ Therefore, the mitochondrial calcium uniporter (MCU) was activated, facilitating Ca^2+^ flow from the cytoplasm (Fluo‐4, green) into mitochondria (Rhod‐2, red) in the PEKK‐L group (Figure 6J,K). Similar findings have been reported in endothelial cells.^[^ ^35^ ^]^ Exogenous addition of spermine enhanced MCU activation in Figure 6J, indicating the ability of spermine to redistribute Ca^2+^. The influx of Ca^2+^ into the mitochondria can increase mitochondrial membrane potential, which provides an explanation for why the biomimetic structure promoted OXPHOS and complex II activity.^[^ ^36^ ^]^

Based on RNA sequencing, the association between MET signaling and Arg2 retrograde translocation was further investigated. MET signaling activated by HGF further promoted the retrograde translocation and the expression of HSP40 and cHSP70 (Figure 6L; Figure S4F, Supporting Information). The expression of PMPCA (mitochondrial processing peptidase), which is involved in cutting MTS, was not affected by the morphology, indicating no change in the rate of MTS cleavage. However, inhibition of MET with SGX‐523 significantly attenuated the subsequent translocation and reprogramming (Figure 6M; Figure S4G–K, Supporting Information). Knockout of Arg2 removed the changes in spermine content, OXPHOS levels, and complex II activity in the PEKK‐L group (Figure 6O–Q). To explore the immunoregulation of the biomimetic PEKK scaffolds after implantation, a mouse dorsal air pouch model was selected due to its sensitivity to early immune responses.^[^ ^5^, ^37^ ^]^ In vivo, PEKK‐L reduced inflammatory responses (Figure 6R) and inhibited M1 polarization (Figure 6S), producing more arginine hydrolyzed metabolites (Figure S4L,M, Supporting Information). Nevertheless, these improvements were significantly weakened after *Arg2* knockout, indicating the importance of Arg2 in regenerative immunoregulation affected by the biomimetic surface design (Figure 6R,S; Figure S4L,M, Supporting Information). Overall, as shown in Figure 6T, the biomimetic design activates MET/Ras/TIAM1 signaling and mediates retrograde translocation of Arg2 with assistance of cHSP70 and HSP40, resulting in increases in spermine and mitochondrial respiration. The retrograde translocation of Arg2 concatenates energy metabolism and MET/Ras/TIAM1 signaling activated by the biomimetic morphology, inducing the ultimate polarization phenotype.

### 2.7 PEKK‐L Promoted M2 Polarization and Enhanced Immune Sensitization

**Figure 7:**
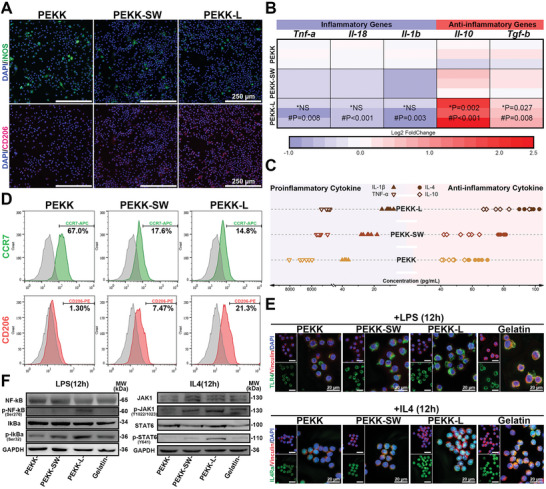
Macrophage polarization and immune sensitization regulated by PEKK‐L in vitro. A) Immunofluorescent staining images of polarization markers of RAW264.7 cells cultured on various scaffolds for 4 days (iNOS, green; CD206, red; DAPI, blue). B) A heat map depicting the fold change in the expression of polarization genes relative to that of the PEKK group (* and # represent PEKK‐L vs PEKK‐SW and PEKK‐L vs PEKK, respectively; *n* = 3). C) The concentration of secreted cytokines of RAW264.7 cells cultured on various scaffolds for 4 days detected by ELISA (*n* = 6). D) Histogram plots comparing the expression of surface markers (CCR7 and CD206) of RAW264.7 cells by flow cytometry. E) Immunofluorescent staining images of TLR4 or IL4Ra and Vinculin (TLR4 or IL4Ra, green; Vinculin, red; DAPI, blue) and F) the expression of downstream proteins of RAW264.7 cells on various scaffolds and gelatin after stimulation of LPS or IL4 for 12 h. Statistical significance was analyzed by ordinary one‐way ANOVA with Tukey's post‐hoc test and respective *P* values are provided.

Clearly, PEKK‐L induced higher expression of CD206 and lower expression of iNOS in RAW264.7 cells compared to other scaffolds (**Figure** **7**A). The expression of anti‐inflammatory genes (*Il‐10*, *Tgf‐b*) in the PEKK‐L group was markedly enhanced, despite no significant difference in the expression of inflammatory genes (*Tnf‐a*, *Il‐18*, and *Il‐1b*) between PEKK‐L and PEKK‐SW (Figure 7B). Moreover, PEKK‐L stimulated the release of anti‐inflammatory cytokines to regulate the microenvironment (Figure 7C). Flow cytometry demonstrated that the biomimetic modification promoted the phenotypic transformation from M1 (CCR7) to M2 (CD206) (Figure 7D; Figure S5A, Supporting Information). A similar phenomenon was observed in OVX‐BMDMs at both the mRNA and cytokine levels (Figure S5B,C, Supporting Information). The efficiency of tissue repair is partly determined by the rate at which macrophages adhering to the implanted materials respond to the immune microenvironment.^[^ ^38^ ^]^ Different stimuli (LPS or IL‐4) at low concentrations were added to polarize cells on the materials, and downstream molecules were detected to evaluate the response efficiency. Spatial limitations on cell adhesion can inhibit immune responses.^[^ ^39^ ^]^ Thus, gelatin with fine cell adhesion ability was used as a control to rule out the effect of differences in cell adhesion alone. The expression of TLR4 or IL‐4Ra was even stronger on the PEKK‐L surface than that on the gelatin after 12 h of stimulation, regardless of the relatively weak adhesion shown by vinculin (Figure 7E). The downstream proteins of classical macrophage polarization pathways were activated separately. Notably, when stimulated by IL4, higher expression of p‐JAK1 and p‐STAT6 was exhibited, indicating superior immune response efficiency of PEKK‐L at the same stimulation concentration (Figure 7F). Similarly, the early responsive gene expression of OVX‐BMDMs increased at ≈3 h in the PEKK‐L group, earlier than that in the other groups at 5 h (Figure S5D, Supporting Information). Additionally, the late responsive genes were highly expressed at a time point (≈5 h) similar to that of the positive control group. Protease K (PK) was used to remove receptors from OVX‐BMDM membranes. The concentrations of TLR4 and IL4R increased in the PEKK‐L group, not only on the cell membranes but also in the cytoplasm (Figure S5E, Supporting Information). In view of this, the biomimetic surface design could effectively coordinate M2 polarization regulation and immune sensitization, resulting in anti‐inflammatory positive feedback and accelerated tissue repair.

### 2.8 PEKK‐L Regulated the Microenvironment to Enhance Bone Regeneration

**Figure 8:**
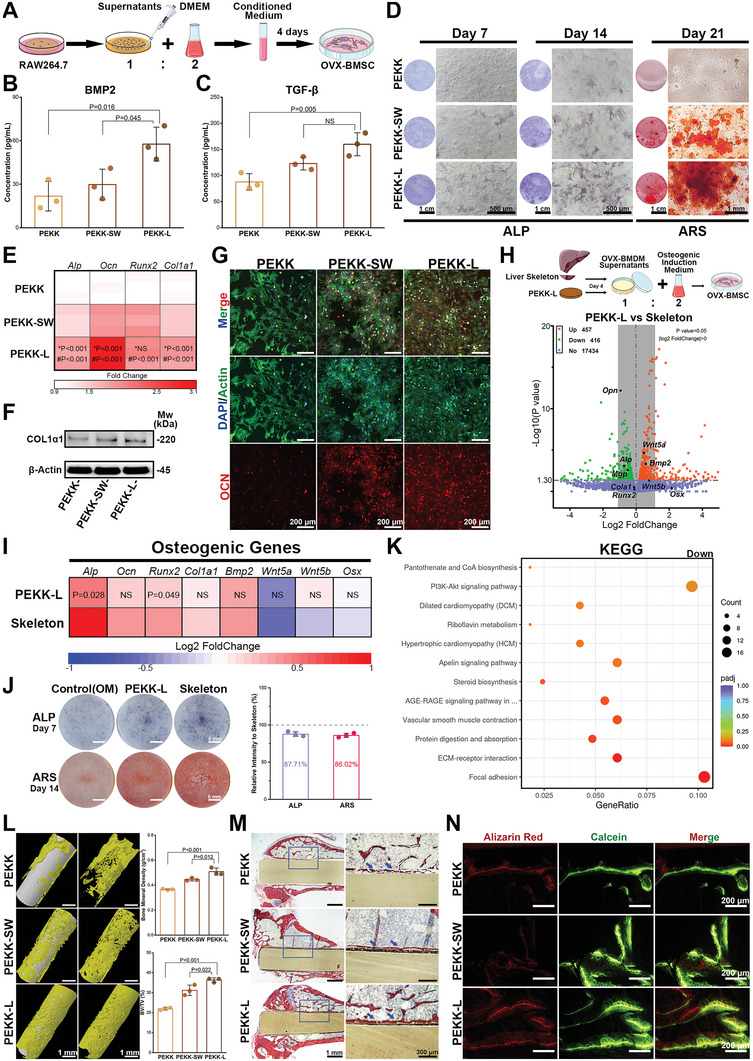
Evaluation of osteogenesis in the microenvironment regulated by various PEKK scaffolds. A) A schematic diagram showing constructed macrophage‐conditioned microenvironment. The concentration of B) BMP2 and C) TGF‐*β* of in the conditioned medium (*n* = 3). D) ALP and ARS staining of OVX‐BMSCs cultured with the RAW264.7 cell‐conditioned medium. E) Heat map depicting the expression of osteogenic genes of OVX‐BMSCs cultured for 14 days with the conditioned medium (* and # represent PEKK‐L vs PEKK‐SW and PEKK‐L vs PEKK, respectively; *n* = 3). Comparison of F) COL1*α*1 and G) OCN (OCN, red; Actin, green; DAPI, blue) of OVX‐BMSCs cultured for 14 days with the conditioned medium. RNA sequencing analysis was performed on OVX‐BMSCs treated with the OVX‐BMDM conditioned medium (*n* = 3). H) A volcano plot of DEGs. I) Heat map depicting the expression of osteogenic genes of OVX‐BMSCs cultured for 7 days with the OVX‐BMDM‐conditioned medium (*n* = 3). J) ALP and ARS staining of OVX‐BMSCs cultured by the OVX‐BMDM‐conditioned medium with the comparison of staining intensity. K) KEGG enrichment analysis of down‐regulated pathways. L) 3D images of micro‐CT after implantation for 8 weeks and quantitative analysis of bone mineral density and bone volume fraction (*n* = 3). M) Van Gieson staining of undecalcified sections (blue arrows indicate bone implant contact). N) Undecalcified sections of sequential polychrome labels for bone (red, Alizarin red, at week 4; green, Calcein, at week 6). Data were analyzed by ordinary one‐way ANOVA with Tukey's post‐hoc test and respective *P* values are provided.

The osteogenesis process is regulated by the immune microenvironment.^[^ ^15^ ^]^ In this study, macrophage‐conditioned medium was used as the mimicking microenvironment (**Figure** **8**A). The conditioned medium of the PEKK‐L group contained more cytokines that are conducive to osteogenesis (Figure 8B,C; Figure S6A, Supporting Information), which promoted osteogenic differentiation of ovariectomized rat bone marrow‐derived mesenchymal stem cells (OVX‐BMSCs) (Figure 8D–G). To investigate the similarity between the microenvironment constructed by PEKK‐L and the liver skeleton bio‐template, RNA sequencing was performed on the OVX‐BMSCs stimulated by different OVX‐BMDM‐conditioned mediums. The analysis revealed both upregulated and downregulated osteogenic genes (Figure 8H). However, there was no significant difference in osteogenesis between the PEKK‐L group and the Skeleton group using RT‐PCR, ALP, and Alizarin red staining (Figure 8I,J), as confirmed by bioinformatics analysis (Figure S6B–D, Supporting Information). The defects lay in focal adhesion and ECM formation (Figure 8K), which might be related to the presence of active components conducive to adhesion of the liver skeleton in the conditioned medium. After implantation in ovariectomized rat femoral defects for 8 weeks, more new bone formation around PEKK‐L was found, presenting complete bridging between PEKK‐L and the adjacent bones and better bone histomorphometry (Figure 8L–N; Figure S6E, Supporting Information).

## 3 Discussion

An effective modification strategy for biological functionalization of bioinert polymer materials is valuable. Recognizing the potential of the liver extracellular skeleton structure to assist in inducing MET signaling, we reproduced the morphological clues of this bio‐template and corresponding biological regulatory functions on the surface of PEKK scaffolds. In previous studies, the structures of micropores, undulations, and grooves constructed on the surface of materials were identified as important morphological factors in the regulation of macrophages.^[^ ^40^ ^]^ Natural tissue structures often contain these multilevel structures and can cascade subsequent biological signals, which are regarded as promising bio‐templates for surface structure modification.^[^ ^41^ ^]^ However, the function of these microstructures is neglected as opposed to the active factors in tissue regeneration.^[^ ^42^ ^]^ MET signaling, closely related to organ regeneration, can trigger intricate biological cascades, resulting in a comprehensive rewiring of gene expression.^[^ ^11^, ^43^ ^]^ The interaction between the *α*6*β*4 integrin and MET implies the molecular‐biological basis of transformation from morphological clues to biological information.^[^ ^44^ ^]^ The interaction and signal transformation induced by the biomimetic morphology may explain the enhanced cell adhesion and regenerative immunoregulation in our study. Hence, functional biomimetic design is an inspiring strategy to improve modification from physical imitation to metaphysical bionics. A skillful utilization of morphology clues is expected to break the choke point of the application of bioinert materials.

The energy metabolism reprogramming in the process of tumor immune escape and invasion induced by MET signaling is enlightening.^[^ ^45^ ^]^ As reported, the activation of Ras signaling drives cellular oxidative respiration, inducing M2 macrophage activation and an anti‐inflammatory microenvironment.^[^ ^46^ ^]^ In view of the involvement of GTP in the activation of the Ras signaling pathway, energy metabolism is essential for MET/Ras/TIAM1 signaling.^[^ ^47^ ^]^ Ca^2+^ signaling has been confirmed to be regulated by TIAM1, which in turn persistently phosphorylates TIAM1 and coordinates mitochondrial dynamics.^[^ ^48^ ^]^ In addition, open conformations of the polypeptide substrate binding domain in HSP70 are regulated through ATP binding and hydrolysis in their nucleotide binding domain, which is closely linked to energy metabolism.^[^ ^49^ ^]^ As an energy‐donating system, the regeneration process of the liver is closely related to energy metabolism as well.^[^ ^50^ ^]^ To a certain extent, this correlation suggests a relationship between the liver extracellular skeleton structure and cellular energy metabolism. Moreover, the osteogenesis process depends on energy metabolism, and energy remodeling in macrophages steers the cell fate of osteoblasts.^[^ ^51^ ^]^

On the one hand, morphology regulation resulted in competition between Arg2 in the arginine hydrolysis pathway and iNOS in the oxidation pathway. On the other hand, the interconversion between ornithine and citrulline maintained a high level of the citrulline product of the arginine oxidation pathway, inhibiting iNOS expression in turn. Other studies have shown the importance of Arg2 in resistance to proinflammatory phenotypes and the proinflammatory cytokine milieu.^[^ ^52^ ^]^ The mitochondrial association of Arg2 may be linked to the degradation of arginine fragments on MTS. Regulated by the biomimetic morphology, Arg2 increased and translocated into the cytoplasm, assisting in the regulation of arginine metabolism. Therefore, unlike the common Arg1, Arg2 can be regarded as an anti‐inflammatory reserve that regulates the polarization process in macrophages. Previous study has highlighted that the combination of LPS and IL10 promotes Arg2 expression rather than Arg1 expression, indicating that Arg2 is essential for the reversal of M2 polarization.^[^ ^53^ ^]^ The situation is similar to that of bioinertness (proinflammatory) combined with the biomimetic design (anti‐inflammatory). Arg2 can increase complex II activity, skewing mitochondrial dynamics and bioenergetics in inflammatory macrophages towards an anti‐inflammatory and oxidative phenotype. The M2 phenotype may be consistent with the preference for OXPHOS.^[^ ^54^ ^]^ Therefore, the strategic utilization of Arg2 as an anti‐inflammatory reserve may be a potentially effective approach to reverse the adverse impact of bioinert materials and orchestrate regenerative immune induction.

## 4 Conclusion

In summary, we found that the liver extracellular skeleton structure could assist the activation of MET signaling in macrophages and induce regenerative immunoregulation. Inspired by this unique surface structure, a biomimetic morphology was prepared on PEKK scaffolds via femtosecond laser etching and sulfonation and successfully reproduced MET signaling. The designated morphological clue was transmitted into macrophages via MET signaling and led to the retrograde translocation of Arg2 from mitochondria into the cytoplasm. The translocation resulted in increases in spermine and mitochondrial respiration, reprogramming energy metabolism and arginine metabolism in macrophages. As for the mechanism of Arg2 retrograde translocation, we proposed the hypothesis of HSP70 spatial binding differences. Moreover, the biomimetic design orchestrated regenerative immunoregulation and boosted osteoporotic osteogenesis. This deliberate strategy simulated morphological clues and corresponding biofunctional signals, achieving functional bionics. Furthermore, the engineered design effectively mobilized the host's anti‐inflammatory reserves, resurging bone regeneration in an inflammatory and pathological microenvironment.

## 5 Experimental Section

### Animals and Cell Culture

All animal experiments were approved by the Animal Health and Experimental Committee of the Ninth People's Hospital and treated under standard laboratory conditions. Animal license number is SCXK (Shanghai) 2012–0007. An osteoporosis rat model was obtained by ovariectomy on 3‐month‐old female Sprague Dawley rats. Arg2^−/−^ C57BL6/J and wild‐type (WT) C57BL6/J mice were purchased from GemPharmatech Co., Ltd.

Cells were cultured in complete DMEM (high glucose Dulbecco's modified Eagle's medium; HyClone, USA) with 10% fetal bovine serum (FBS) (Gibco, USA) at 37 °C and 5% CO_2_ levels. The primary OVX‐BMSCs were isolated from ovariectomized rats 3 months after ovariectomy. Rats were euthanized in a CO_2_ chamber, and bone marrow was collected from femurs and tibias to isolate cells. After reaching 80% confluence, cell passage could be performed, and the cultured cells from passage 3 were used for further experiments. OVX‐BMDMs were similarly derived from ovariectomized rats. The bone marrow was plated in 10 cm petri dishes in complete DMEM with 10% FBS and M‐CSF (20 ng mL^−1^; Beyotime, China) and incubated for 6 days. OVX‐BMDMs were then collected for subsequent experiments. Arg2^−/−^ BMDMs were obtained from Arg2^−/−^ mice in a similar way. RAW264.7 cells were cultured according to the protocol suggested by the American Type Culture Collection.

### Immunofluorescent Staining

Cells were fixed in 4% paraformaldehyde for 30 min and permeabilized by 0.1% Triton‐X for 15 min. The tissue blocks were fixed, embedded, and sliced. The samples were blocked with 1% BSA for 30 min and incubated with primary antibodies overnight at 4 °C. A secondary antibody was added and incubated for 2 h at room temperature. 4′,6‐diamidino‐2‐phenylindole pihydrochloride (DAPI) was used to stain nuclei. Immunofluorescent staining images were captured by confocal laser scanning microscopy (CLSM; Leica, Germany) or fluorescence microscopy (Zeiss, Germany).

### Immunoblotting

Cells were collected and lysed in RIPA buffer with a protease inhibitor cocktail and 1 mm PMSF on ice. The supernatants were obtained after centrifugation at 12 000× g for 15 min at 4 °C and were detected for protein concentrations by the BCA kit (Abcam, USA). Fifteen micrograms sample per well was loaded on 4–16% gradient Bis‐Tris gels (Invitrogen, USA). After electrophoresis, proteins were transferred to PVDF membranes. The membranes were blocked and incubated with primary antibodies overnight. Blots were further incubated with the HRP‐conjugated secondary antibody and ECL reagent and captured by gel imaging analysis (UVITEC, Britain).

### Real‐Time Polymerase Chain Reaction

Cells were extracted by TRIzol. Complementary DNA (cDNA) was synthesized from total RNA by reverse transcriptase M‐MLV (Takara, Japan) after extraction and centrifugation. Quantitative gene analysis was performed using SYBR Premix Ex Taq II (Takara, Japan) and an RT‐PCR analyzer (Roche, Switzerland). Relative gene quantitation was assessed by the 2‐^ΔΔCT^ method. Primers are listed in Table S1 (Supporting Information).

### Liver Decellularization

The liver tissue was derived from 8‐week‐old WT C57BL6/J mice. The liver tissue blocks (8 × 2 mm) were cut from the surface of livers of euthanized mice. The blocks were rinsed with ultrapure water for 24 h and decellularized for 2 days with 0.2% sodium dodecyl sulfate (SDS). Prior to cell experiments, the liver extracellular skeleton was sterilized with 0.1% peracetic acid and rinsed with phosphate‐buffered saline (PBS). The liver extracellular skeleton was further lyophilized for 24 h. For Skeleton‐Flat, prior to lyophilization, the acellular tissue blocks were physically pressed into thin tablets to remove surface characteristics. The similarity of the two skeleton components was demonstrated by a Raman Spectrometer (Renishaw, Britain) and an ELISA kit (R&D Systems, USA) for hepatocyte growth factor (HGF). The decellularization was evaluated by a DNA content kit (TIANGEN, China), hematoxylin and eosin (H&E) staining, and DAPI staining. The morphology was observed by field‐emission scanning electron microscopy (TESCAN, Czech Republic).

### MET Signaling Assessment

The expression of polarization‐related genes (*Tnf‐a*, *Il‐1b*, *Il10*, and *Tgf‐b*) stimulated by HGF and a hepatocyte growth factor receptor (MET) inhibitor (SGX‐523) for 4 days was assessed by RT‐PCR. The expression of proteins MET and p‐MET in RAW264.7 cells cultured on the two liver skeleton samples for 4 days was detected by immunoblotting. In order to explore the relationship between MET, polarization, and morphology, HGF antibody (HGF^ab^, 1 µg mL^−1^; R&D Systems, USA) and MET antibody (MET^ab^, 1 µg mL^−1^; R&D Systems, USA) were, respectively, added to weaken the influence of HGF and MET. The polarization markers (iNOS and CD206; Santa Cruz) were stained and captured by CLSM. The expression of proteins MET and p‐MET (ABclonal, China) in RAW264.7 cells cultured on PEKK and titanium scaffolds for 4 days was detected by immunoblotting. The change in macrophage polarization and constructed osteogenic microenvironment was assessed by RT‐PCR and ELISA kits for BMP2 and TGF‐*β* (R&D Systems, USA).

### Biomimetic Scaffold Preparation

Commercially available PEKK rods (Polymics, USA) were prepared with dimensions of Φ20 × 1 mm^3^ for material characterization and in vitro cell experiments. Samples with dimensions of Φ10 × 1 mm^3^ and Φ2 × 5 mm^3^ were employed in the mouse dorsal pouch model and the rat bone repair model in vivo, respectively. To prepare biomimetic scaffolds, samples were first etched via the femtosecond laser direct writing system using a compact ytterbium‐doped diode pumped ultrafast amplified laser at the center wavelength of 1030 nm, which has a pulse width of 500 fs.^[^ ^55^ ^]^ The laser beam was focused by an objective lens with a numerical aperture of 0.50 (RMS20X‐PF, Olympus). The moving speed was 500 µm s^−1^ and the moving direction was perpendicular to the laser beam. A single pulse energy of ≈0.5 µJ was used, and the repetition rate was 300 kHz. The etched samples were ultrasonically cleaned in acetone, ethanol, and ultrapure water sequentially and then underwent 5 min of sulfonation with 80% sulfuric acid and 24 h of hydrothermal reaction (denoted as PEKK‐L). For PEKK‐SW, the samples were immersed in 98% sulfuric acid solution for 5 min and then were hydrothermal reacted for 24 h to remove the residual acid.

### Material Characterization

SEM images and element analysis were conducted by FE‐SEM. The surface morphology and roughness (arithmetic average error, Ra) were assessed by a 3D optical profiler (Bruker, Germany). The surface nanotopography and elastic modulus were characterized by atomic force microscopy (Bruker, Germany) in tapping mode. The groups and hydrophilicity were detected by a Fourier transform infrared spectrometer (FTIR; Thermo, USA) and a droplet shape analyzer (KRUSS, Germany). After various scaffolds were soaked in simulated body fluid for 1, 3, 5, 7, and 14 days, respectively, pH values of the solution were recorded by a pH analyzer (LEICI, China). The scaffolds were immersed in bovine serum albumin solutions (100 µg mL^−1^) at 37 °C for 4 h. After washing off unabsorbed proteins, adsorbed BSA was isolated by immersion in 2% SDS and sonication to detect concentration.

### Material Biocompatibility and Functional Bionics

RAW264.7 cells or OVX‐BMDMs were seeded on scaffolds at an initial concentration of 5 × 10^4^ per well and cultured for 1 and 4 days, respectively. The cells were further cultured in fresh medium with the addition of 10% CCK‐8 (Dojindo, Japan) for 30 min at 37 °C and detected at 450 nm absorbance by a spectrophotometer (TECAN, Switzerland). RAW264.7 cells were stained with calcein‐AM and propidium iodide to assess cell activity. The morphology of RAW264.7 cells cultured on scaffolds for 3 days was observed by SEM and pseudocolored red by Photoshop software. The cytoskeleton of OVX‐BMDMs were stained with F‐actin (Abcam, USA). The functional bionics of MET signaling were first assessed by the analysis of differentially expressed genes related to the Ras signaling pathway via RNA sequencing. The expression of MET, p‐MET, and TIAM1 in RAW264.7 cells on scaffolds under or without stimulation of HGF and SGX‐523 was detected by immunoblotting.

### RNA Sequencing Analysis and Central Carbon Metabolism Analysis

For RNA sequencing, RAW264.7 cells were cultured on various scaffolds (initially 5 × 10^4^ per well) for 3 days. Total RNA was collected using the TRIzol reagent. For metabolomics analysis, RAW264.7 cells on scaffolds were collected, rinsed, centrifuged, and then saved in liquid nitrogen. The gene expression and metabolomics analyses were performed at Shanghai Biotree Biotech. The recurring upregulated genes of PEKK‐L compared with PEKK and PEKK‐SW constituted a new gene cluster. The functional enrichment analysis of the obtained gene cluster was evaluated by the Gene Ontology (GO) database and the Kyoto Encyclopedia of Genes and Genomes (KEGG) database. Metabolite changes were shown in the central carbon metabolic pathway. Fold changes in the expression of genes were displayed. The KEGG and biological process enrichment analyses of differentially expressed genes were performed. The gene set variation analysis (GSVA) of biological processes and signaling pathways related to osteogenesis was further assessed.

### Mitochondrial Respiration Assessment

The oxygen consumption rates (OCRs) were detected by Seahorse XFe96 analyzer (Agilent, USA). RAW264.7 cells were cultured on various scaffolds for 3 days and transferred to microplates (4 × 10^4^ cells per well) for another 10 h before assay. Oligomycin (1 µm), carbonyl cyanide 4‐ (trifluoromethoxy) phenylhydrazone (FCCP, 0.9 µm) and rotenone/antimycin A (Rot/Ant A, 0.5 µm) were sequentially added. The basal respiration, ATP production, and maximal respiration were calculated according to OCR curves. The ADP/ATP ratio was assessed by an ADP/ATP assay kit (Dojindo, Japan). The mitochondrial morphology of RAW264.7 cells cultured on scaffolds for 3 days was stained with MitoBright LT (Dojindo, Japan) at 37 °C for 30 min and observed by CLSM. Quantitative assessments of mitochondrial morphology were analyzed via MiNA (Mitochondrial Network Analysis) and MitoAnalyzer plugins of ImageJ software. The expression of genes related to mitochondrial fission and fusion (*Drp1* and *Mfn2*) was detected by RT‐PCR. The production of intracellular ROS was labeled with 2′,7′‐dichlorodihydrofluorescein diacetate (DCFH‐DA; Dojindo, Japan) for 30 min. Mitochondrial superoxide and membrane potential were stained with MitoSOX Red and MT‐1 (Dojindo, Japan), respectively. The fluorescence intensity of stained images was analyzed by ImageJ software. Total ATP and glycolytic ATP were detected by a glycolysis/OXPHOS assay kit (Dojindo, Japan) with or without oligomycin (2.5 µm) to assess OXPHOS levels. After extracting proteins from samples and adjusting protein concentration to 5.5 mg mL^−1^, the activity of complex I enzyme and complex II enzyme was, respectively, assayed via the Complex I Enzyme Activity Assay Kit and the Complex II Enzyme Activity Microplate Assay Kit according to the protocols (Abcam, USA).

### Mitochondrial Isolation

RAW264.7 cells or OVX‐BMDMs were collected and added to mitochondrial separation reagent (Beyotime) with 1 mM PMSF. The cell suspension was left on ice for 15 min and then homogenized in a glass homogenizer with 30 strokes. The homogenates were centrifuged (600× g) for 10 min at 4 °C, and the obtained supernatant was further centrifuged (11 000× g) for 10 min. The pellet was saved and considered mitochondrial proteins. The remaining supernatant was then centrifuged (12 000× g) for 10 min and collected as cytoplasmic proteins.

### Metabolite Analysis

Cells cultured on various scaffolds for 3 days were collected and homogenized on ice. After centrifugation (12 000× g) for 10 min at 4 °C, the supernatant was transferred into a 10 kDa MWCO spin column and centrifuged at 10 000× g for 20 min. The filtrate was collected for further detection. The concentrations of arginine, fumarate, ornithine, and citrulline were, respectively, assessed by Arginine assay Kit, Fumarate Detection Kit, Ornithine Assay Kit, and Citrulline Assay Kit according to the provided protocols (Abcam, USA). For lactate and urea, the culture medium of RAW264.7 cells was collected and diluted by ultrapure water to a suitable concentration. The final concentration was, respectively, detected by a lactate assay kit (Dojindo, Japan) and a urea assay kit (Abcam, USA). In addition, the culture medium of RAW264.7 cells was de‐proteinized by 10 kDa columns and centrifuged (10 000× g, 4 °C for 10 min). The ultra‐filtrate was collected to analyze nitrite content according to the protocol of Griess reagent (Abcam, USA). For spermine, the culture medium was homogenized in equal volumes of cold 1 m HClO_4_. The samples were analyzed by high‐performance liquid chromatography (HPLC) on a column of cation‐exchange resin in a Hitachi L6000 high speed liquid chromatograph.

### Arginase Activity and Ornithine Decarboxylase Activity

Arginase activity was assessed by measuring urea content. The cell suspension was homogenized and distinguished between mitochondrial proteins and cytoplasmic proteins by mitochondrial isolation. The activity of arginase in mitochondria was compared with that in the cytoplasm. The activity of ornithine decarboxylase was detected using an ornithine decarboxylase ELISA kit (Biomatik).

### Immunoelectron Microscopy

Briefly, cell samples were fixed and dehydrated via high‐pressure freezing fixation and freeze‐substitution. The samples were then placed onto a formvar‐coated 200 mesh nickel grid stabilized with carbon (Ted Pella). The grids were rinsed with DPBS and incubated with an anti‐Arg2 primary antibody and the corresponding secondary antibody conjugated with 18 nm gold particles. After incubation, samples were rinsed, air dried, and captured by a transmission electron microscope (Thermo, USA).

### Molecular Docking

The structures of Arg2 and HSP70 were obtained from the PDB database (4HZE and 1S3X). Molecular docking analysis was performed by HDOCK software with flexible docking methods. The interaction analysis was displayed by the software programs PyMOL and Maestro. The structure of HSP70 and HSP40 was obtained from the PDB database (5NRO).

### Lentivirus‐shArg2 Transfection

RAW264.7 cells (10^5^ per well) were seeded in a 12‐well plate overnight. Lv‐shArg2 and control shRNA lentiviral particles (Lv‐NC) were added to a mixture of complete medium with polybrene (5 µg mL^−1^) for cell culture for 12 h according to the protocols (Santa Cruz, USA). The cells were further cultured in fresh complete medium (without polybrene and lentiviral particles) and selected by puromycin dihydrochloride (5 µg mL^−1^). The resistant colonies were collected to seed on the liver skeleton to detect the expression of genes related to arginine metabolism.

### Ca^2+^ Distribution

RAW264.7 cells cultured on scaffolds for 3 days were stained with Fluo‐4 AM (37 °C for 30 min; Dojindo, Japan) and Rhod‐2 AM (4 °C for 30 min; Dojindo, Japan) to label Ca^2+^ in the cytoplasm and mitochondria, respectively.^[^ ^56^ ^]^

### Polarization and Immune Sensitization

RAW264.7 cells or OVX‐BMDMs were seeded on scaffolds for 4 days. The expression of genes (*Tnf‐a, Il‐18, Il‐1b, Nos2, Il‐4, Il‐10, Vegf*, and *Tgf‐b*) was detected by RT‐PCR. The polarization markers iNOS (Santa Cruz, USA) and CD206 (Santa Cruz, USA) were captured by CLSM. After collecting the supernatant, the concentrations of cytokines (IL‐1*β*, TNF‐*α*, IL‐4, and IL‐10) were, respectively, detected by ELISA kits (Absin, China). A flow cytometer (Millipore, USA) was used to assess the polarization of RAW264.7 cells stained by allophycocyanin (APC)‐conjugated CCR7 and phycoerythrin (PE)‐conjugated CD206. The data were analyzed by FlowJo software. The concentrations of cytokines in OVX‐BMDMs were detected by Luminex technology with the assistance of Shanghai Universal Biotech Co., Ltd. For immune sensitization, RAW264.7 cells or OVX‐BMDMs were seeded on scaffolds for 12 h and further stimulated by polarization stimuli at low concentrations (LPS, 20 ng mL^−1^, Sigma, USA; IL‐4, 5 ng mL^−1^, R&D Systems, USA). RAW264.7 cells were stained with Vinculin antibody (ABclonal, China) and TLR4 Alexa Fluor 488 (Santa Cruz, USA) or IL‐4Ra Alexa Fluor 488 (Santa Cruz, USA). The expression of downstream proteins was assessed by immunoblotting with primary antibodies against NF‐kB, p‐NF‐kB, IkBa, p‐IkBa, JAK1, p‐JAK1, STAT6, p‐STAT6 (ABclonal, China). Total RNA of OVX‐BMDMs was collected after stimulation for 1, 3, 5, and 7 h, respectively, to assess expression of early responsive genes (*Tlr4*, *Cxcl2*, *Il4ra*, and *Mrc1*) and late responsive genes (*Il1b*, *Il6*, *Il10*, and *Tgfb1*). The concentrations of TLR4 (Novus, USA) and IL4R (Abbexa, Britain) in OVX‐BMDMs of RAW264.7 cells after stimulation for 12 h were detected by ELISA. Protease K (0.1, 1, and 10 µg mL^−1^) was added to remove membrane receptors to measure intracellular content.

### Osteogenesis Evaluation

The macrophage‐conditioned medium was prepared from the supernatant of macrophages on scaffolds for 4 days and mixed with DMEM at a 1:2 ratio. The concentrations of BMP‐2 and TGF‐*β* in macrophage‐conditioned medium were monitored by ELISA kits (R&D Systems, USA). The expression of osteogenesis genes, protein Col1a1 (Cell Signaling Technology, USA) and protein OCN (Cell Signaling Technology, USA) in OVX‐BMSCs cultured in macrophage‐conditioned medium for 14 days was assessed by RT‐PCR, immunoblotting, and immunofluorescent staining. Moreover, at days 7 and 14, OVX‐BMSCs were fixed and stained with alkaline phosphatase reagent (Beyotime) or Alizarin red dye (Cyagen) for 15 min at 37 °C, respectively.

### Surgical Procedure

An air pouch was formed on the backs of C57BL/6 mice by injecting sterile air.^[^ ^5b^ ^]^ The scaffold samples were inserted into the pouches of WT mice and Arg2^−/−^ mice under anesthesia. Four days after implantation, the skin covering the implants was cut and fixed. The skin sections were stained with H&E. The polarization markers (iNOS and Arg2, Santa Cruz, USA) of macrophages in the fibrous layer were labeled. The inflammatory cells in the air pouch were rinsed with PBS and collected to detect the concentrations of nitrite and urea.

For osseointegration assessment, cylindrical scaffolds were implanted parallel to the long axis in the femurs of ovariectomized rats. Alizarin red and calcein dyes were injected to mark new bone formation after 4 and 6 weeks, respectively. After implantation for 8 weeks, the femurs were collected and scanned by micro‐CT. In addition, the femurs were embedded and sliced. Alizarin red and calcein staining images were captured by a fluorescence microscope. The sections were stained with Van Gieson dye (Solarbio, China) as well.

### Statistical Analysis

The data in the figures were displayed as the mean ± standard deviation. Statistical differences were analyzed with a one‐way analysis of variance (ANOVA) with Tukey's post‐hoc test by OriginPro software.

## Conflict of Interest

The authors declare no conflict of interest.

## Supporting information

Supporting Information

## Data Availability

The data that support the findings of this study are available from the corresponding author upon reasonable request.
