# Supplementary material for: Liver‐Inspired Polyetherketoneketone Scaffolds Simulate Regenerative Signals and Mobilize Anti‐Inflammatory Reserves to Reprogram Macrophage Metabolism for Boosted Osteoporotic Osseointegration
Source: Adv Sci (Weinh). 2023 Jul 3;10(25):2302136. doi: 10.1002/advs.202302136 (PMC10477864; doi:10.1002/advs.202302136)
Supplement: Supplementary file 1 — Supporting Information [file ADVS-10-2302136-s001.pdf]

ADVANCED SCIENCE
Open Access

Supporting Information

for *Adv. Sci.*, DOI 10.1002/advs.202302136

Liver-Inspired Polyetherketoneketone Scaffolds Simulate Regenerative Signals and Mobilize Anti-Inflammatory Reserves to Reprogram Macrophage Metabolism for Boosted Osteoporotic Osseointegration

Hao Gu, Yuhui Zhu, Jiawei Yang, Ruixue Jiang, Yuwei Deng, Anshuo Li, Yingjing Fang, Qianju Wu, Honghuan Tu, Haishuang Chang, Jin Wen and Xinquan Jiang**

Supporting Information

Liver-inspired Polyetherketoneketone Scaffolds Simulate Regenerative Signals and Mobilize Anti-inflammatory Reserves to Reprogram Macrophage Metabolism for Boosted Osteoporotic Osseointegration

Hao Gu, Yuhui Zhu, Jiawei Yang, Ruixue Jiang, Yuwei Deng, Anshuo Li, Yingjing Fang, Qianju Wu, Honghuan Tu, Haishuang Chang, Jin Wen, and Xinquan Jiang**

This file includes:

Figure S1 to S6
Table S1

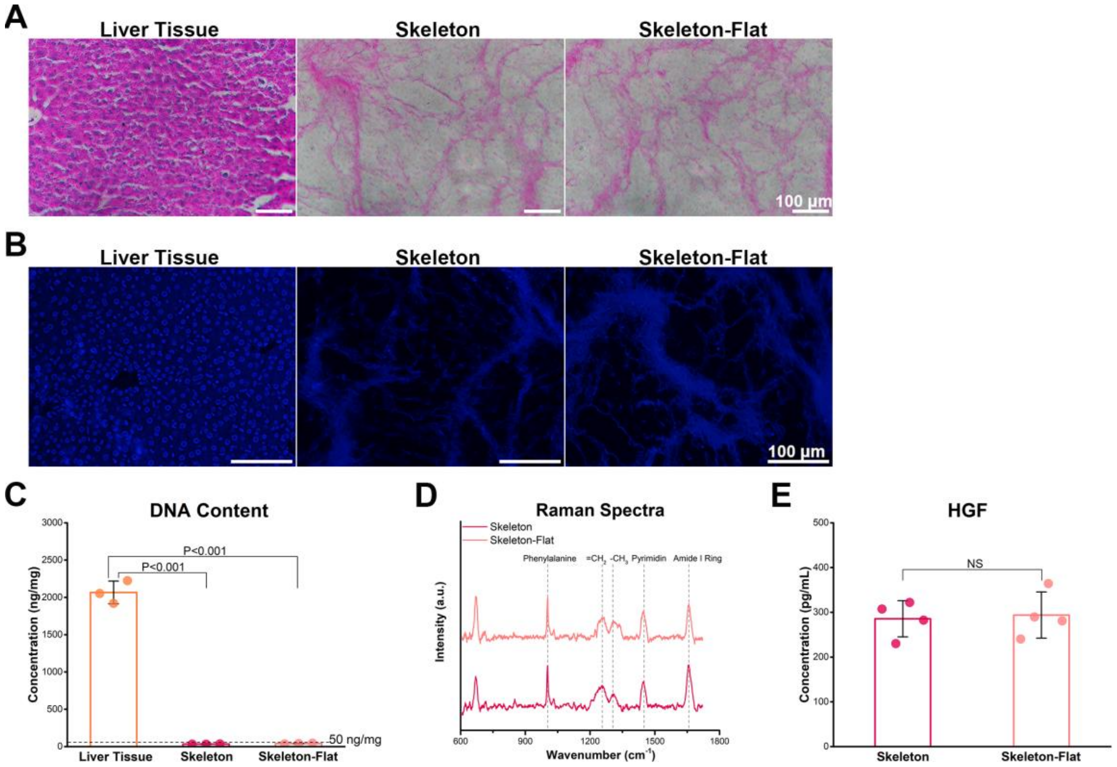

Figure S1. Preparation and characterization of the liver extracellular skeleton. A) Hematoxylin and eosin (HE) staining, B) DAPI staining and C) DNA quantification of samples of the Liver Tissue group, the Skeleton group and the Skeleton-Flat group confirming nuclei depletion (error bars, means ± SD; n = 3). D) Raman spectra of the Skeleton sample and the Skeleton-Flat sample. E) The content of HGF in the two liver extracellular skeleton samples detected by ELISA (error bars, means ± SD; n = 4). Data were analyzed by C) ordinary one-way ANOVA with Tukey's post-hoc test, E) two-tailed t-test with Welch's correction and respective P values are provided.

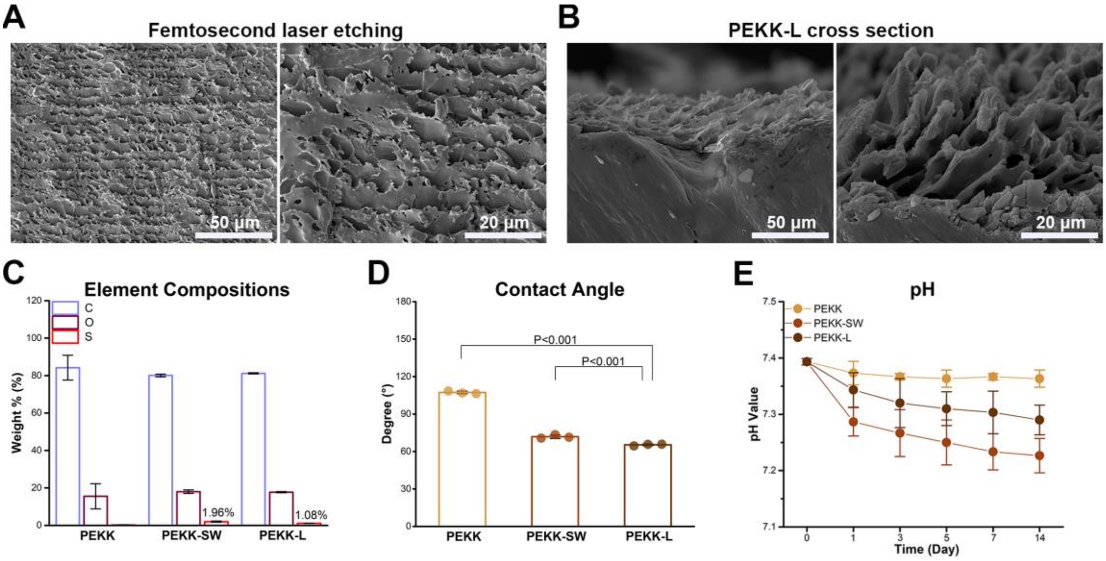

Figure S2. Characterization of various PEKK scaffolds. A) SEM images of the surface of PEKK scaffolds modified by femtosecond laser etching. B) SEM images of cross section of the biomimetic scaffolds. C) The element compositions of various PEKK scaffolds (error bars, means ± SD; n = 3). D) Contact angles of water droplets on the surface of various PEKK scaffolds (error bars, means ± SD; n = 3). E) The changes in pH value of the solution after various scaffolds soaking in simulated body fluid for different lengths of time (error bars, means ± SD; n = 3). Statistical significance was analyzed by ordinary one-way ANOVA with Tukey’s post-hoc test and respective P values are provided.

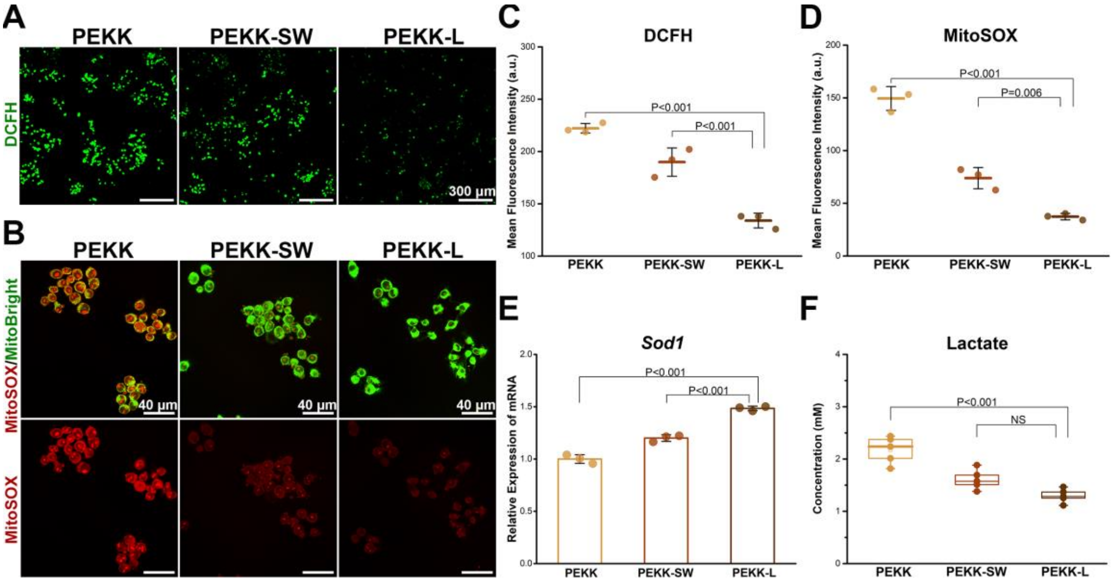

Figure S3. Evaluation of antioxidative system of macrophages regulated by various PEKK scaffolds. A) Immunofluorescent images staining with DCFH for intracellular reactive oxygen species (ROS) of RAW264.7 cells cultured on scaffolds for 3 days. B) Representative fluorescent images staining with MitoSOX for mitochondrial ROS of RAW264.7 cells cultured on various scaffolds for 3 days. Quantification of C) DCFH staining and D) MitoSOX staining (error bars, means ± SD; n = 3). E) Expression of antioxidase gene *Sod1* in RAW264.7 cells cultured on various scaffolds (error bars, means ± SD; n = 3). F) Comparison of RAW264.7 cells for levels of lactate regulated by various scaffolds (lower and upper box boundaries, line inside box and lower and upper lines represent 25th and 75th percentiles, median, minimum and maximum respectively; n = 5). Statistical significance was analyzed by ordinary one-way ANOVA with Tukey’s post-hoc test and respective P values are provided.

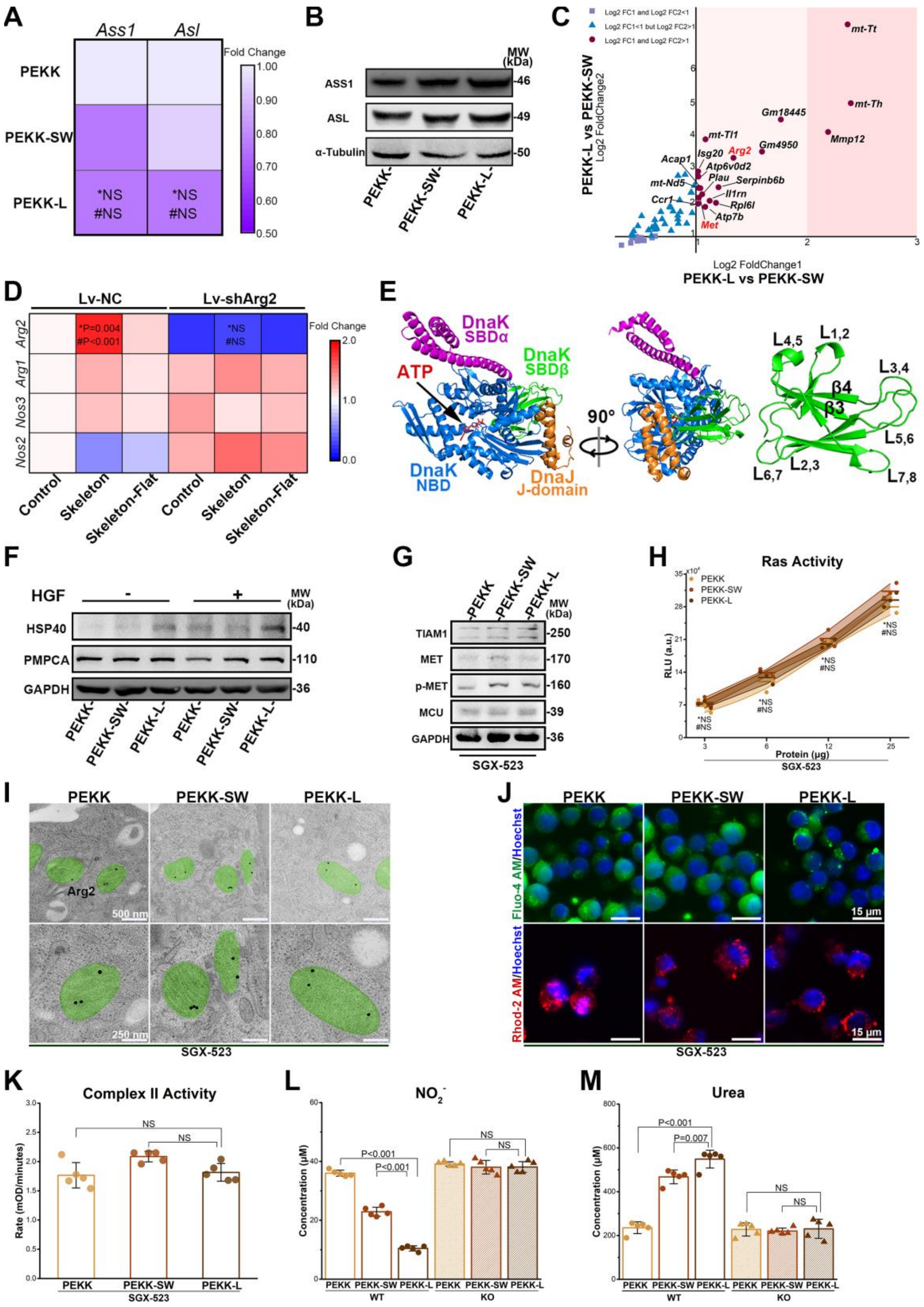

Figure S4. The effect of MET signaling suppression on Arg2 translocation and metabolic reprogramming. A) Heat map depicting the expression of *Ass1* and *Asl* in RAW264.7 cells on scaffolds for 3 days (n = 3; * and # represent PEKK-L versus PEKK-SW and PEKK-L versus PEKK respectively). B) Protein expression of ASS1 and ASL. C) The expression of genes in the obtained cluster in Figure 4A. D) Heat

map depicting the expression of genes related to arginine metabolism of RAW264.7 cells transfected with lentivirus-negative control or lentivirus-shArg2 on two types of liver skeleton cultured for 3 days (n = 3; * and # represent Skeleton versus Skeleton-Flat and Skeleton versus Control respectively). E) Conformations of HSP70 (NBD, blue; SBDα, purple; SBDβ, green) and HSP40 (orange). F) Expression of HSP40 and mitochondrial processing peptidase (PMPCA) treated with or without HGF. The changes in G) expression of selected proteins and H) Ras activity (n = 3; * and # represent PEKK-L versus PEKK-SW and PEKK-L versus PEKK respectively) treated with SGX-523. I) Immunoelectron microscopy of mitochondria (green) and Arg2 (black) treated with SGX-523. J) Immunofluorescent staining of cytoplasmic Ca^{2+} (green) and mitochondrial Ca^{2+} (red) with SGX-523 treatment. K) Complex II activity treated with SGX-523 (n = 5). L, M) Metabolite (NO^- and urea) levels in the tissue fluid surrounding scaffolds after implantation for 3 days in wild type and *Arg2*$^{-/-}$ mice (n = 5). Data were analyzed by ordinary one-way ANOVA with Tukey’s post-hoc test and respective P values are provided.

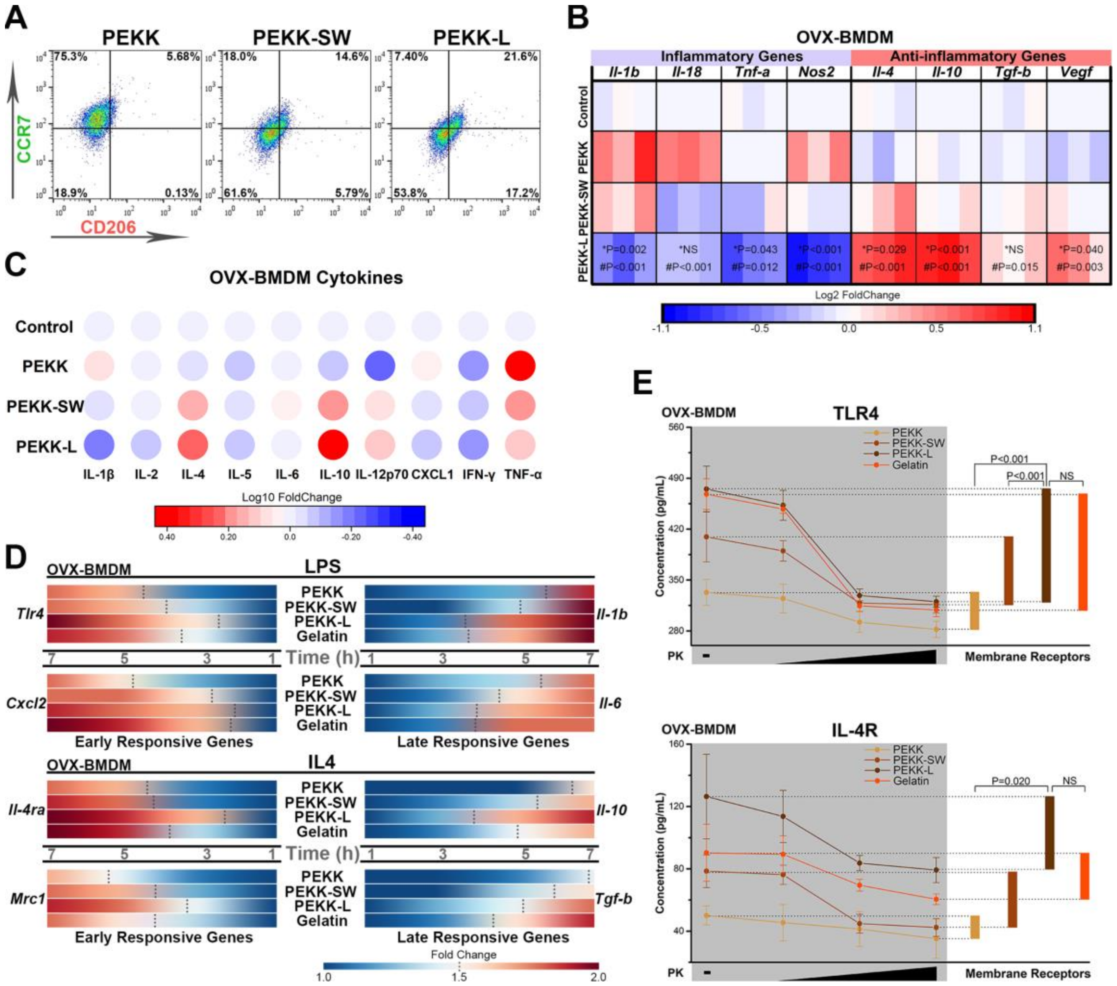

Figure S5. OVX-BMDM polarization and immune sensitization regulated by PEKK-L *in vitro*. A) Representative dot images of surface markers (CCR7 and CD206) of RAW264.7 analyzed by flow cytometry in Figure 7D. Heat maps depicting the fold changes B) in the expression of polarization genes (n = 3; * and # represent PEKK-L versus PEKK-SW and PEKK-L versus PEKK respectively) and C) in concentrations of secreted cytokines detected by Luminex technology (n = 3) of OVX-BMDMs cultured on various scaffolds for 4 days relative to that of the Control group. D) Heat maps depicting the fold changes in the expression of selected genes of OVX-BMDMs on scaffolds and gelatin after stimulation for 1, 3, 5, and 7 hours respectively relative to that of 1 h stimulation (n = 3; dotted lines mark the 1.5 fold change). E) The concentrations of TLR4 and IL4R of OVX-BMDMs on various scaffolds and gelatin after stimulation for 12 hours detected by ELISA (error bars, means ± SD; n = 3). Protease K of different concentrations (0, 0.1, 1 and 10 μg/mL) was used to remove membrane receptors to determine the content of intracellular receptor proteins. Statistical significance was analyzed by ordinary one-way ANOVA with Tukey's post-hoc test and respective P values are provided.

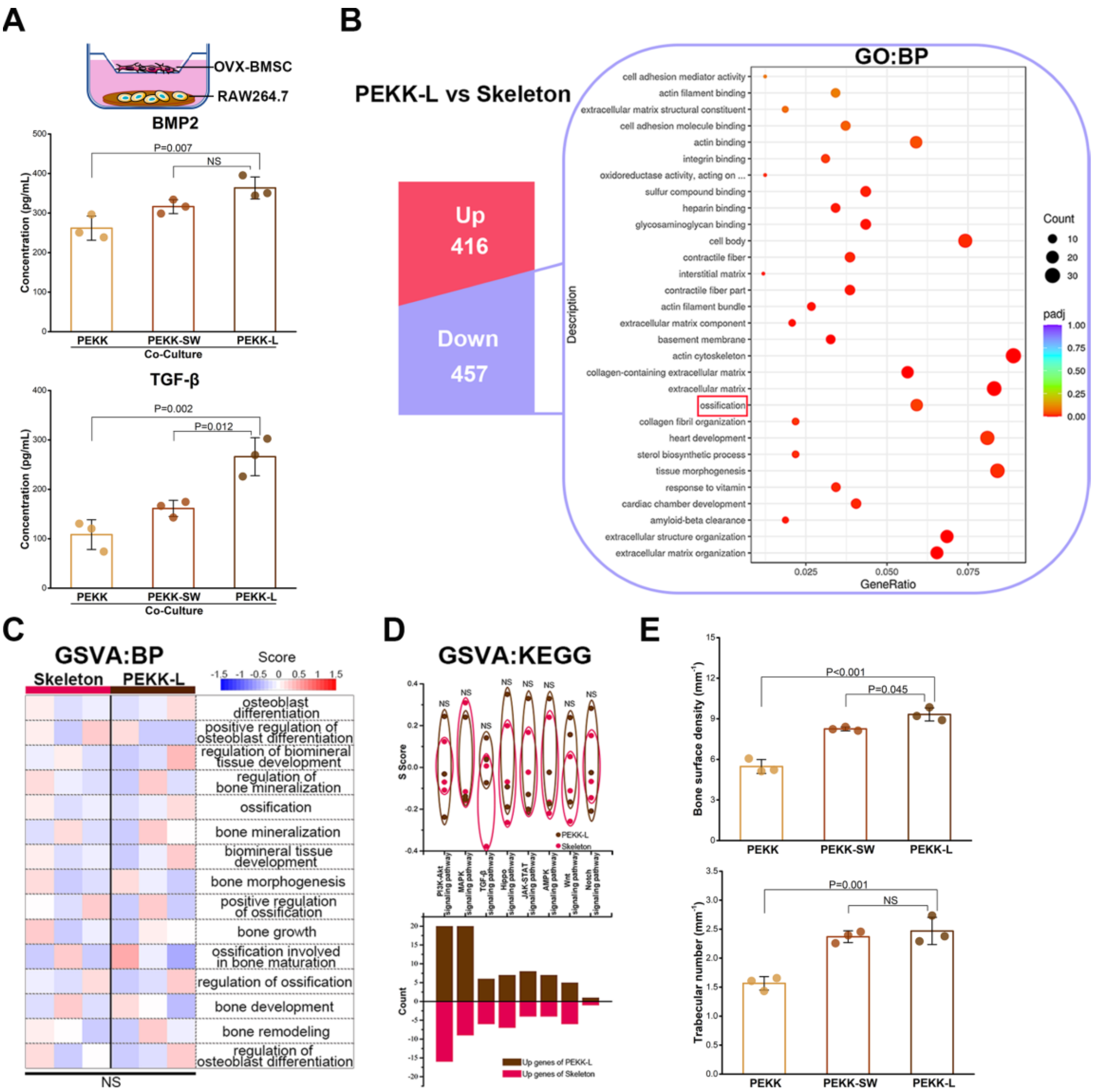

Figure S6. Evaluation of osteogenic microenvironment created by macrophages on various PEKK scaffolds. A) A schematic diagram showing constructed co-cultured macrophage-conditioned microenvironment by Transwell and the concentrations of BMP2 and TGF-β of the co-culture microenvironment regulated by RAW264.7 cells on various PEKK scaffolds (error bars, means ± SD; n = 3). B) Biological process enrichment analysis of differentially expressed genes and gene set variation analysis (GSVA) of C) biological process and D) signaling pathways related to osteogenesis with enrichment of differential genes in Figure 8H. E) Quantitative analysis of bone surface density and trabecular number according to micro-CT data in Figure 8L (error bars, means ± SD; n = 3). Statistical significance was analyzed by ordinary one-way ANOVA with Tukey's post-hoc test and respective P values are provided.

Table S1. Primer sequences for the genes observed in this study.

Gene	Forward primer	Reverse primer
Actin	CTTTCTACAATGAGCTGCGTG	ATGGCTGGGGTGTTGAAGG
Alp	GGGACTGGTACTCGGACAAT	GGCCTTCTCATCCAGTTCAT
Gapdh	GTCGTGGAGTCTACTGGTGTC	GAGCCCTTCCACAATGCCAAA
Runx2	ACAACCACAGAACCACAAG	TCTCGGTGGCTGGTAGTGA
Ocn	GGTGCAGACCTAGCAGACACCA	AGGTAGCGCCGGAGTCTATTCA
Il-18	TGGCCGACTTCACTGTACAAC	TGGGGTTCACTGGCACTTTG
Col1a1	CATGTTCAGCTTTGTGGACCT	GCAGCTGACTTCAGGGATGT
Il-1b	TGGAGAGTGTGGATCCCAAG	GGTGCTGATGTACCAGTTGG
Tgf-b	CAGTACAGCAAGGTCCTTGC	ACGTAGTAGACGATGGGCAG
Vegf	GTCCCATGAAGTGATCAAGTTC	TCTGCATGGTGATGTTGCTCTCTG
Il-10	GAGAAGCATGGCCCAGAAATC	GAGAAATCGATGACAGCGCC
Sod1	CACTCTAAGAAACATGGTGG	GATCACACGATCTTCAATGG
Drp1	CCGGGAATGACCAAAGTACC	TGGGATTACTGATGAACCGAAGA
Mfn2	CCAACTCCAAGTGTCCGCTC	GTCCAGCTCCGTGGTAACATC
Asl	TCTTCCCAGGGTACACACAC	GGCCCCAAAGTTCAGTTCTG
Ass1	ACCATCCTTTACCACGCTCA	ACCTGCACCTTCCCTTCTAC
Tnf-a	CTGAACTTCGGGGTGATCGG	GGCTTGTCACTCGAATTTTGAGA
Il-4	ACAGCCTCACAGAGCAGAAGACT	TGTGTTCTTGGAGGCAGCAA
Bmp2	GCTCCACAAACGAGAAAAGC	AGCAAGGGGAAAAGGACACT
Wnt5a	ATTCTTGGTGGTCGCTAGGT	TGTACTGCATGTGGTCCTGA
Wnt5b	AGACTGGCATCAAGGAATGC	GTCTCTCGGCTGCCTATCTG
Osx	CCTCTCGACCCGACTGCAGATC	AGCTGCAAGCTCTCTGTAACCATGAC
Nos2	GCCACCAACAATGGCAACA	GTACCGGATGAGCTGTGAATT
Nos3	AGGGGAACAAGCCCAGTAGT	AATTCGCCAATGACAAGACG
Arg1	GTGAAGAACCCACGGTCTGT	CTGGTTGTCAGGGGAGTGTT
Arg2	GGATCCAGAAGGTGATGGAA	AGAGCTGACAGCAACCCTGT
Tlr4	CCGCTTTCACCTCTGCCTTCAC	ACCACAATAACCTTCCGGCTCTTG
Cxcl2	GAAGTCATAGCCACTCTCAAGG	CCTCCTTTCCAGGTCAGTTAGC
Il-6	ATAGTCCTTCCTACCCCAATTTCC	GATGAATTGGATGGTCTTGGTCC
Mrc1	AGACGAAATCCCTGCTACTG	CACCCATTCGAAGGCATTC
Il-4ra	TGGATCTGGGAGCATCAAGGT	TGGAAGTGCGGATGTAGTCAG
